# DNA Topoisomerase 3α Is Involved in Homologous Recombination Repair and Replication Stress Response in *Trypanosoma cruzi*

**DOI:** 10.3389/fcell.2021.633195

**Published:** 2021-05-13

**Authors:** Héllida Marina Costa-Silva, Bruno Carvalho Resende, Adriana Castilhos Souza Umaki, Willian Prado, Marcelo Santos da Silva, Stela Virgílio, Andrea Mara Macedo, Sérgio Danilo Junho Pena, Erich Birelli Tahara, Luiz Ricardo Orsini Tosi, Maria Carolina Elias, Luciana Oliveira Andrade, João Luís Reis-Cunha, Glória Regina Franco, Stenio Perdigão Fragoso, Carlos Renato Machado

**Affiliations:** ^1^Laboratório de Genética Bioquímica, Departamento de Bioquímica e Imunologia, Instituto de Ciências Biológicas, Universidade Federal de Minas Gerais, Belo Horizonte, Brazil; ^2^Laboratório de Biologia Molecular e Sistêmica de Tripanossomatídeos, Instituto Carlos Chagas, Fundação Oswaldo Cruz (FIOCRUZ), Curitiba, Brazil; ^3^Laboratório de Ciclo Celular, Centro de Toxinas, Resposta Imune e Sinalização Celular, Instituto Butantan, São Paulo, Brazil; ^4^Laboratório de Biologia Molecular de Leishmanias, Departamento de Biologia Celular e Molecular e Bioagentes Patogênicos, Faculdade de Medicina de Ribeirão Preto, Universidade de São Paulo (USP), Ribeirão Preto, Brazil; ^5^Laboratório de Biologia Celular e Molecular, Departamento de Morfologia, Instituto de Ciências Biológicas, Universidade Federal de Minas Gerais, Belo Horizonte, Brazil; ^6^Departamento de Medicina Veterinária Preventiva, Escola de Veterinária, Universidade Federal de Minas Gerais, Belo Horizonte, Brazil

**Keywords:** DNA topoisomerase 3α, homologous recombination, replication stress, *Trypanosoma cruzi*, DNA damage, DNA repair, dormancy

## Abstract

DNA topoisomerases are enzymes that modulate DNA topology. Among them, topoisomerase 3α is engaged in genomic maintenance acting in DNA replication termination, sister chromatid separation, and dissolution of recombination intermediates. To evaluate the role of this enzyme in *Trypanosoma cruzi*, the etiologic agent of Chagas disease, a topoisomerase 3α knockout parasite (TcTopo3α KO) was generated, and the parasite growth, as well as its response to several DNA damage agents, were evaluated. There was no growth alteration caused by the TcTopo3α knockout in epimastigote forms, but a higher dormancy rate was observed. TcTopo3α KO trypomastigote forms displayed reduced invasion rates in LLC-MK2 cells when compared with the wild-type lineage. Amastigote proliferation was also compromised in the TcTopo3α KO, and a higher number of dormant cells was observed. Additionally, TcTopo3α KO epimastigotes were not able to recover cell growth after gamma radiation exposure, suggesting the involvement of topoisomerase 3α in homologous recombination. These parasites were also sensitive to drugs that generate replication stress, such as cisplatin (Cis), hydroxyurea (HU), and methyl methanesulfonate (MMS). In response to HU and Cis treatments, TcTopo3α KO parasites showed a slower cell growth and was not able to efficiently repair the DNA damage induced by these genotoxic agents. The cell growth phenotype observed after MMS treatment was similar to that observed after gamma radiation, although there were fewer dormant cells after MMS exposure. TcTopo3α KO parasites showed a population with sub-G1 DNA content and strong γH2A signal 48 h after MMS treatment. So, it is possible that DNA-damaged cell proliferation due to the absence of TcTopo3α leads to cell death. Whole genome sequencing of MMS-treated parasites showed a significant reduction in the content of the multigene families DFG-1 and RHS, and also a possible erosion of the sub-telomeric region from chromosome 22, relative to non-treated knockout parasites. Southern blot experiments suggest telomere shortening, which could indicate genomic instability in TcTopo3α KO cells owing to MMS treatment. Thus, topoisomerase 3α is important for homologous recombination repair and replication stress in *T. cruzi*, even though all the pathways in which this enzyme participates during the replication stress response remains elusive.

## Introduction

The protozoan *Trypanosoma cruzi* is the etiological agent of Chagas disease, also known as American trypanosomiasis. This disease, endemic in Latin America, has been spreading to other continents due to migratory flow. Around 8 million people worldwide are infected by this parasite (Echeverria and Morillo, 2019). *T. cruzi* belongs to the family Trypanosomatidae, which includes the genera *Trypanosoma* and *Leishmania*. During its complex life cycle, *T. cruzi* alternates between an insect vector and a vertebrate host. Epimastigotes and metacyclic trypomastigotes are life forms found in triatomine insects, whereas amastigotes and bloodstream trypomastigotes are present in the mammalian host. Epimastigotes and amastigotes are proliferative forms, while metacyclic and bloodstream trypomastigotes are infective forms. Additionally, these developmental stages also differ in the cell shape, organelle position, and metabolism (Jimenez, 2014).

The DNA damage response (DDR) is a signal transduction pathway responsible for detecting all kinds of DNA damage and replication stress, in which several proteins work in a coordinated way to halt cell cycle progression and activate DNA repair mechanisms (Ciccia and Elledge, 2010). Two pivotal kinases in DDR are ATM (Ataxia Telangiectasia Mutated) and ATR (Ataxia Telangiectasia and Rad3-Related), which are activated by DNA damage and phosphorylate serine and threonine residues in their protein targets. ATM responds to DNA double-strand breaks (DSB) and promotes homologous recombination repair. ATR is activated by the presence of long tracts of single-stranded DNA (ssDNA), functioning as the major kinase in the replication stress response. Even though ATM and ATR have specific functions and substrates, there is an interconnection between their cellular responses (Cimprich and Cortez, 2008).

DNA topoisomerases are conserved enzymes essential to solve topological problems generated during DNA metabolism and gene expression, performing their functions via transesterification reactions (Cuya et al., 2017). The reaction catalyzed by the topoisomerases does not change the DNA sequence and is not dependent on sequence recognition, so these enzymes can act on any region of the DNA with torsional problems (Pommier et al., 2010; Pommier et al., 2016).

DNA topoisomerases are classified as type I and type II. Type I enzymes cleave DNA single strands, while type II topoisomerases form dimers to cleave DNA double strands, in an ATP-dependent manner. Each topoisomerase type can be subdivided into two subfamilies: IA, IB, IIA, and IIB (Wang, 2002). Each subfamily plays specific roles within the cell, allowing precise coordination of the topological state of DNA throughout the cell cycle (Pommier et al., 2016).

The topoisomerases type IA can relax the negative supercoiling that occur during DNA replication elongation and DNA transcription. Furthermore, due to their ability to catalyze single-strand decatenation, they can act at the termination of DNA replication, chromosome segregation, and dissolution of recombination repair intermediates. This indicates the role of these enzymes in maintaining genomic stability (Pommier et al., 2016). Two genes encoding topoisomerases of the subfamily IA were identified in *Escherichia coli*. Prokaryotic proteins are named topoisomerase 1 and topoisomerase 3. Eukaryotes have only topoisomerase 3, and in the model eukaryotes, there are two isoforms of this enzyme, called topoisomerase 3α and topoisomerase 3β (Capranico et al., 2017).

At the end of the homologous recombination repair, the dissipation of the double Holliday junctions is necessary, which allows the separation between the two chromosomes. In humans, DNA topoisomerase 3α forms a complex with BLM (Bloom syndrome protein), a RecQ DNA helicase, and with RMI1/2 factors, which can dissolute the double Holliday junctions, generating non-crossover products (Yang et al., 2010). It is known that the physical interaction among these proteins is required to promote the dissolution of these structures. The BLM helicase is responsible for the convergent branch migration of the junctions. The catalytic action of the helicase provides a substrate of single-strand DNA, the hemicatenanes, which can be processed by topoisomerase 3α. The RMI1/2 binding modulates the topoisomerase decatenation activity, making the dissolution of the Holliday junctions more efficient (Bizard and Hickson, 2014; Bocquet et al., 2014). In summary, DNA topoisomerase 3α contributes to genomic stability by preventing chromosome rearrangement and allowing the completion of homologous recombination (Capranico et al., 2017).

Genomic analysis in the TriTrypDB has shown that trypanosomatids have a complete set of the various types of DNA topoisomerases in their genome. In these parasites, the enzymes of the subfamily IA are topoisomerase IA, topoisomerase 3α, and topoisomerase 3β. The topoisomerases of this subfamily are phylogenetically well conserved and are compartmentalized in the nucleus and mitochondria of trypanosomatids (Balaña-Fouce et al., 2014). Studies in *T. brucei* showed that topoisomerase 3α is important for the antigenic variation mediated by homologous recombination (Kim and Cross, 2010). Despite its relevant role in eukaryotic cells, there is no information about topoisomerase 3α in *T. cruzi*. Here, we analyze the effects of topoisomerase 3α depletion in different life forms of *T. cruzi*. Our results suggest that this enzyme is necessary to repair DSBs generated after gamma radiation, since knockout parasites were unable to proliferate after irradiation. Furthermore, the topoisomerase 3α absence influenced the fork replication recovery after cisplatin (Cis), hydroxyurea (HU), and methyl methanesulfonate (MMS) treatments. Indeed, knockout parasites showed a significant reduction in the content of the multigene families DFG-1 and RHS (retrotransposon hot spot), and possible telomere shortening after MMS treatment. Together, these results imply that topoisomerase 3α is necessary to maintain genomic stability in *T. cruzi*.

## Materials and Methods

### Cell Cultures and Growth Conditions

Epimastigote forms of *T. cruzi* Dm28c strain were cultivated in liver infusion tryptose (LIT) medium pH 7.4 supplemented with 10% inactivated fetal bovine serum (Gibco) and 1% streptomycin/penicillin (Invitrogen), at 28°C. For the culturing of knockout parasites, 300 μg ml^–1^ of hygromycin B (Invitrogen) and neomycin (G418 sulfate – Gibco) antibiotics were added to the culture medium. Rhesus monkey kidney monolayers cells (LLC-MK2) (Hull et al., 1962) were maintained in 10% Dulbecco’s modified Eagle’s medium (10% DMEM; Sigma Aldrich), containing 10% fetal bovine serum (Gibco), 200 U ml^–1^ of penicillin, and 200 μg L^–1^ of streptomycin sulfate. Metacyclic trypomastigotes obtained from axenic cultures of *T. cruzi* at stationary phase were used to initiate parasite intracellular life cycle in LLC-MK2 cells. Infection was performed in DMEM supplemented with 2% fetal bovine serum (Gibco), 200 U ml^–1^ penicillin, and 200 μg ml^–1^ streptomycin sulfate (2% DMEM). LLC-MK2 cultures were washed daily with PBS^+/+^ buffer (NaCl 0.134 M, KCl 2.7 mM, Na_2_HPO_4_ 10 mM, KH_2_PO_4_ 1.8 mM, Ca^2+^ 0.9 mM, and Mg^2+^ 0.49 mM) to remove remaining epimastigotes. Released tissue-culture trypomastigotes (TCTs) were purified as described previously (Andrews et al., 1987) and used to maintain parasite intracellular life cycle and to perform all experiments involving these cells.

### Construction of Topoisomerase 3α Knockout Parasites

To generate knockout parasites, the regions comprising nucleotides 67 up to 401 (5′CDS3α–334 bp of length) and 2,391 up to 2,747 (3′CDS3α–356 bp of length) of the coding sequence of the TcTopo3α gene (GenBank: AY850132–2,766 bp of length) were amplified from *T. cruzi* Dm28c genomic DNA by polymerase chain reaction (PCR). The 5′CDS3α and 3′CDS3α were amplified using the primers described in Supplementary Table 1. The 5′CDS3α amplicon was digested with *Kpn*I and *Sal*I restriction enzymes (New England Biolabs), while 3′CDS3α amplicon was digested with *BamH*I and *Xba*I restriction enzymes (New England Biolabs). Both digested amplicons were cloned into pTc2KO-neo or pTc2KO-hygro vectors, which carry neomycin or hygromycin B-resistance gene (Pavani et al., 2016). The complete deletion cassettes were denominated as pNEOΔTopo3α and pHYGROΔTopo3α. The pNEOΔTopo3α cassette was amplified by PCR, and it was transfected into wild-type (WT) *T. cruzi* epimastigotes using a Gene Pulser II electroporation system (Bio-Rad). The selection occurred in LIT medium with 500 μg ml^–1^ of G418 until the death of the control parasites. To confirm the insertion of the cassette in the correct loci, a PCR reaction with DNA of transfected parasites was performed using a forward primer located inside the neomycin resistance gene and a reverse primer located 434 bp downstream of the TcTopo3α gene (InNeo and DS3α primers sequences, respectively, are shown in Supplementary Table 1). After that, these parasites were transfected with pHYGROΔTopo3α cassette as described above. The selection occurred in LIT medium with 500 μg ml^–1^ of G418 and hygromycin B until the control parasites’ death. The correct insertion of the cassette was tested in the selected parasites by PCR using the primers InHygro forward located inside the hygromycin B resistance gene and DS3α reverse primer.

### Pulsed-Field Gel Electrophoresis and Southern Blot Analysis

To confirm the absence of the topoisomerase 3α gene in the mutant parasites, the chromosomes from WT and TcTopo3α KO cells were separated by PFGE using the LKB Pulsaphor (Pharmacia). Exponential growth parasites were recovered from LIT medium and washed with PBS buffer twice. Then the cells were resuspended in PSG buffer (44 mM NaCl, 57 mM Na_2_HPO_4_, 3 mM KH_2_PO_4_, 55 mM glucose) and mixed with an equal volume of 1% low-melting point agarose. To prepare these agarose blocks containing intact chromosomes, 2 × 10^7^ parasites for each block were used. The blocks were incubated with a lysis solution (0.5 M EDTA pH 9, 1% sarkosyl and 0.5 mg/ml proteinase K) at 50°C for 50 h and then stored in this same solution at 4°C. Before the electrophoresis, the blocks were incubated three times with 50 mM EDTA pH 8 for 1 h. The blocks were subjected to PFGE on 1.2 % agarose gel in 0.5× TBE buffer (Tris-borate 44.5 mM, boric acid 44.5 mM, 1 mM EDTA) at 10°C. Chromosomes from yeast *Hansenula wingei* (1.05 to 3.13 Mbp) were used as a molecular weight marker (CHEF DNA Size Markers-Bio-Rad). The PFGE was carried out in a constant voltage of 100 V for 135 h with five phases of pulses (N/S, E/W): 90 s for 30 h, 200 s for 30 h, 350 s for 25 h, 500 s for 25 h, and 800 s for 25 h. Following electrophoresis, the gel was stained with ethidium bromide (0.5 μg ml^–1^) and photographed with L-Pix photodocumentation system (Loccus Biotecnologia). The Southern blot analysis was performed according to standard protocols (Sambrook and Russell, 2001). To detect specific DNA sequences, DNA bands were transferred to nylon membranes and hybridized with forward and reverse probes for topoisomerase 3α, hygromycin, and neomycin genes. All probes were radioactively labeled with α-[P^32^]-dCTP using the Nick Translation Labeling Kit (Invitrogen) following the manufacturer’s recommendations (probes sequences are shown in Supplementary Table 1).

In order to assess the role of TcTopo3α gene in genomic stability maintenance, another Southern blot analysis was performed. To that end, genomic DNA (2 μg) from WT and Topo3α KO cells was treated with CviQI, HpaII, AluI, and HhaI restriction endonucleases at 37°C for 24 h. Digestion products were resolved by gel electrophoresis (0.7% agarose; 1X TAE buffer-Tris-Base 0.04 M, EDTA 0.05 M, Acetic acid 5,71% – at 40 V; for 16 h). DNA was transferred to Hybond-N^+^ membranes (GE Life Sciences) and probed with a 300-bp fragment containing telomeric repeat TTAGGG (Van der Ploeg et al., 1984). Hybridization was carried out at 60°C using Amersham^TM^ AlkPhos Direct Labeling and Detection System with Amersham^TM^ CDP-Star^TM^ Detection Reagent (GE Life Sciences).

### Epimastigote Growth Curves

To verify whether topoisomerase 3α gene is important during DSB repair in *T. cruzi*, the growth profile of WT parasites was compared with TcTopo3α KO parasites after gamma radiation exposure. The parasites were irradiated with a dose of 1,541 Gy h^–1^ for 19 min and 28 s using a cobalt (^60^Co) irradiator located at Laboratório de Irradiação Gama (CDTN/CNEN, UFMG). Additionally, parasites were subjected to replication stress in growth curves performed with 100 μM of cisplatin (Cis) in PBS buffer for 1 h at 28°C; 20 mM of hydroxyurea (HU) in LIT medium at 28°C for 24 h, and 1.5 mM of methyl methanesulfonate (MMS) in PBS buffer for 1 h at 28°C. After each treatment, the cells were washed with PBS to remove the drug. Then the parasites were incubated in a fresh LIT medium at 28°C. For ATR inhibition analysis, parasites were kept in LIT medium containing 5 mM caffeine or 10 μM VE-821 (Sigma). All growth curves were initiated at a density of 1 × 10^7^ cells ml^–1^ with cells in log phase growth. The cells were counted daily until the control cells reached stationary phase. The number of parasites was determined using a cytometry chamber and vital dye erythrosine. For all treatments tested, it was performed at least three independent experiments in triplicate.

All treatments were followed by CellTrace CFSE (Thermo Fisher) labeling as previously described (Resende et al., 2020). Briefly, 2 × 10^7^ epimastigotes ml^–1^ were incubated for 20 min at 28°C with 10 mM CFSE in PBS buffer, protected from light. The excess of CFSE was quenched with five volumes of LIT medium for 5 min. After that, cells were centrifuged at 3,000 × *g* for 10 min and resuspended in fresh LIT medium at a concentration of 1 × 10^7^ cells ml^–1^. Aliquots from CFSE-stained epimastigote cultures were collected until 144 h for all treatments, fixed overnight with 4% paraformaldehyde (PFA) at 4°C and analyzed by flow cytometry to assess fluorescence intensity. FACSCan or FACSCalibur flow cytometers (Benckton-Dickson) were used for data collection, and 10,000 events for each condition were analyzed using the software FlowJo VX.

### Mammalian Cell Infection and Immunostaining for Invasion Rates and Amastigote Growth Curves

For cell infection assays, 4 × 10^4^ LLC-MK2 cells were suspended in 10% DMEM and added onto 13-mm round glass coverslips inserted into each well of a 24-well plate. Plated cells were then incubated at 37°C with 5% CO_2_ for 24 h before infection with purified trypomastigotes from WT and TcTopo3α KO parasites, previously labeled with CellTrace CFSE. For trypomastigote CFSE labeling, parasites were incubated for 20 min at 37°C with 10 mM CFSE in PBS buffer. The excess of CFSE was quenched with 2% DMEM for 5 min. After that, cells were centrifuged at 3,000 × *g* for 10 min and resuspended with five volumes of 2% DMEM. Infection was performed (protected from light) at a multiplicity of infection (MOI) of 50 for 1 h in 2% DMEM. Afterward, cells were washed five times with PBS^+/+^ and re-incubated in 2% DMEM for additional 24, 48, 72, and 96 h, until overnight fixation with 4% PFA at 4°C temperature in which samples were stored until processed for immunofluorescence. The coverslips with attached cells were washed three times with PBS^+/+^, incubated for 20 min with PBS containing 2% BSA (PBS/BSA), and processed for an inside/outside immunofluorescence invasion assay as previously described (Andrews et al., 1987). Briefly, extracellular parasites were immunostained with rabbit anti-*T. cruzi* polyclonal antibody (Andrade and Andrews, 2004) in a 1:500 dilution in PBS/BSA for 1 h at room temperature, washed and labeled with Alexa Fluor-546 conjugated anti-rabbit IgG antibody (Thermo Fisher Scientific) in a proportion of 1:500 in PBS/BSA for 45 min. After that, DNA from host cells and parasites was stained for 1 min with 0.1 μM DAPI (4′,6-diamidino-2-phenylindole, dihydrochloride – Sigma) in PBS, mounted, and examined on a Zeiss Axio Vert.A1 microscope equipped with an AXIOCAM ICM1 camera controlled by the ZEN Image Software (Zeiss).

### Analysis of Cell Cycle Progression by Flow Cytometry

In order to evaluate the cell cycle alterations due to replication stress, WT and TcTopo3α KO parasites were analyzed by flow cytometry after treatment with Cis (100 μM), HU (20 mM), or MMS (1.5 mM). Cells in log phase growth were treated with the drugs, as described above, and samples were collected each 24 h for cell cycle progression assay. Thus, 1 × 10^7^ cells ml^–1^ were harvested by centrifugation at 2,500 × *g* for 10 min, washed with PBS buffer, and fixed in EtOH 70% at −20°C for at least 16 h. For DNA staining, the cells were washed and resuspended in PBS buffer containing 10 μg ml^–1^ RNAse A (Invitrogen) and 10 μg ml^–1^ propidium iodide (BD Pharmingen) and incubated in the dark at 37°C for 30 min. The data were collected at FACSCan or FACSCalibur flow cytometer (Benckton-Dickson) from 10,000 events and analyzed with the software FlowJo VX.

### Preparation of Protein Extracts and Western Blot Analysis

To measure the level of DNA damage in WT and Topo3α KO parasites after treatment with Cis (100 μM), HU (20 mM), or MMS (1.5 mM), a Western blot assay was performed. To prepare the protein extracts, 1 × 10^8^ cells from each culture were harvested by centrifugation at 2,500 × *g* for 10 min and was washed twice with PBS buffer. The cells were lysed with 50 μl of sample buffer 2× (100 mM Tris-HCl pH 6.8, 4% sodium dodecyl sulfate, 0.2% bromophenol blue, 20% glycerol, 200 mM dithiothreitol). Then the samples were sonicated in the Sonic Dismembrator Model 500 (Fisher Scientific) apparatus at 30% maximum amplitude for five cycles of 20 s, with an interval of 20 s. After that, the samples were boiled for 10 min and stored at −20°C. Protein concentration was determined using the Bradford method. To separate the proteins according to their size, an SDS-PAGE was performed. Both the 5% stacking gel and the 15% separation gel were prepared following standard protocols (Sambrook and Russell, 2001). The protein extracts were thawed and boiled for 5 min. The electrophoresis was carried out at 125 V for 2 h with 15 μg of each protein extract. Then, proteins were electroblotted onto a polyvinylidene difluoride membrane using the Trans-Blot^®^ SD Semi-Dry Transfer Cell (Bio-Rad) for 1 h at 300 mA. The membranes were blocked by incubation in blocking solution, which consists of TBST buffer (150 mM NaCl, 20 mM Tris pH 7.5 and 0.1% Tween 20) with 5% non-fat dry milk for 2 h under agitation. After that, the membranes were washed three times with TBST buffer for 5 min. Then, the membranes were incubated with the primary antibody anti-γH2A (1:3,000) produced in rabbit and kindly provided by Dr. Richard McCulloch’s group. Alternatively, the membranes were incubated with the primary antibody anti-α-tubulin (1:10,000) produced in mouse (Sigma). For both antibodies, the incubation was carried out in blocking solution overnight under agitation. The membranes were washed three times with TBST for 5 min. Then, the membranes were incubated with secondary antibodies (1:5,000) conjugated with peroxidase, anti-rabbit IgG (Sigma Immuno Chemicals), or anti-mouse IgG (Sigma) for 1 h under agitation. The membranes were washed three times with TBST for 5 min and revealed using Immobilion^TM^ Western Chemiluminescent HRP Substrate (Millipore) system and ImageQuant LAS 500 (GE) apparatus.

### 5-Bromo-2′-deoxyuridine Native Detection Assay

To verify the involvement of topoisomerase 3α in the replication stress response after HU (20 mM) and MMS (1.5 mM) treatments, a BrdU native detection assay was performed, following the protocol used by Dias and collaborators, with small modifications (Dias et al., 2019). Both treated and control parasites were incubated with 100 μM of 5-bromo-2′-deoxyuridine (BrdU) for 24 h to allow its incorporation into DNA. Then the parasites were harvested by centrifugation at 2,000 × *g* for 5 min, washed with PBS buffer, and fixed using 4% paraformaldehyde diluted in PBS for 10 min at room temperature. Then parasites were spread out onto slides (previously treated with 0.1% poly-L-lysine), washed with PBS buffer, and permeabilized with 0.2% Triton X-100 for 10 min at room temperature. As a positive control for BrdU incorporation, slides containing samples from WT and Topo3α KO untreated parasites were subjected to DNA denaturation using 2.5 M HCl for 20 min. Then all samples were washed, and accessible BrdU was detected using α-BrdU-rat (Abcam) diluted 1:250 in blocking solution (4% bovine serum albumin in PBS) for 3 h at room temperature, followed by incubation for an additional 3 h with secondary antibody Alexa Fluor 555-conjugated goat anti-rat (Thermo Scientific) diluted 1:1,000 in blocking solution. After that, the slides were washed repeatedly using PBS buffer. VECTASHIELD^®^ Mounting Medium with DAPI (Vector Labs) was used to be the anti-fade mounting solution and to stain DNA content. Images were captured using Olympus BX51 fluorescence microscope coupled with a digital camera (XM10, Olympus) and were analyzed using Olympus-Cell F software. Differences in parasite ssDNA foci were measured using ImageJ software.

### Genome Sequencing and Read Mapping

To evaluate the DNA content after MMS treatment a whole genome sequencing (WGS) was performed at Glasgow Polyomics. WT and Topo3α KO cultures containing 1 × 10^7^ cells ml^–1^ were cultivated in LIT medium supplemented with fetal bovine serum and 1 mM MMS. Every day, for 5 days, cells were centrifuged at 3,000 × *g* for 5 min at room temperature and resuspended in fresh medium with 0.75 mM MMS. After five generations, cells were counted and washed three times with PBS buffer. DNA extraction was performed using Qiagen Blood and Cell Culture DNA Mini Kit (Cat:13323) following manufacturer protocol for cell culture with an incubation time in proteinase K (kit provided) of 3 h. As control, a non-treated culture was submitted to the same steps of cultivation and DNA extraction, using the same volume of PBS instead of MMS in the medium. The DNA purity and quantity were determined using NanoDrop^TM^ 1000 UV–Vis spectrophotometer, and a paired-end library with mean fragment size of 800 bp was constructed using Nextera^TM^ DNA kits (Illumina Inc.). The library was sequenced on Illumina NextSeq^TM^ 500 platform with generation of 75 bp reads.

Reads from each WGS library were quality checked using FastQC^1^, and high-quality reads were selected using Trimmomatic.v.0.33 (Bolger et al., 2014), with a minimum threshold of phred quality of 30 and a minimum length of 50 nucleotides. High-quality reads were mapped in the 41 chromosomes from *T. cruzi* CL Brener Non-Esmeraldo-like genome v.46 (Aslett et al., 2009) using BWA-mem (Li and Durbin, 2010). The mapped reads were filtered by mapping quality 30 using SAMtools v1.1 (Li et al., 2009) for the CCNV estimations, and no mapping quality filter was applied to the estimations of the read depth of each multigene family (see the *Estimation of Genomic Alterations by Comparative Genomics Approaches* section). The absence of a mapping quality filter is important to better estimate read depth in *T. cruzi* multigene families. To evaluate the deletion of TcTopo3α (TcCLB.508851.170), the read depth of CL Brener Chr18 region that contains this gene was visualized in IGV v2.6.2 (Thorvaldsdóttir et al., 2013). The reads from WGS library were submitted to the NCBI Sequence Read Archive (SRA), accession codes: SRR13754248, SRR13754250, SRR13754249, and SRR13754247 (BioProject: PRJNA702660). A Dm28c non-TcTopo3α-KO WGS read library SRR7592211 was used as a control for TcCLB.508851.170 gene presence in this analysis.

### Estimation of Genomic Alterations by Comparative Genomics Approaches

The potential impact of MMS treatment in SNP generation, ploidy, and gene copy number alterations in TcTopo3α KO cells was also assessed in MMS-treated (MM1 and MM2) and untreated (NT1 and NT2) isolates. To estimate alterations in gene and chromosome copies, the read depth of each position in each chromosome was determined with BEDTools genomecov v2.16.2 (Quinlan and Hall, 2010), and the coverage of each gene was obtained using *in-house* Perl scripts that extract the gene coverages using CDS coordinates in GFF files. Chromosomal copy number variation (CCNV) was evaluated as described by Reis-Cunha and collaborators (Reis-Cunha et al., 2018). Briefly, for each chromosome, genes with outlier coverages were excluded with iterative Grubb’s tests (*p* < 0.05), and the median RDC on non-outlier genes in each chromosome was normalized by the genome coverage and assumed as the chromosomal somy. Next, to evaluate segmental duplications/deletions, the coverage of each position of each chromosome was normalized by the genome coverage, and MM1 and MM2 coverages were summed and subtracted from the sum of NT1 and NT2. Hence, values above zero correspond to a higher copy number in MMS-treated isolates, and values below zero correspond to higher copy numbers in MMS-untreated isolates. The image representing these segmental duplications was generated in R (^2^ R development 2020), using the genoPlotR library^3^. The gene coordinates were obtained from *T. cruzi* CL Brener Non-Esmeraldo-like GFF v.46 (Aslett et al., 2009). The impact of MMS treatment in the copy number of *T. cruzi* multigene families DGF-1, MASP, RHS, TcMUC, and trans-sialidase was also evaluated. The read depth of each gene from each family in MMS-treated and untreated parasites was obtained, and the median coverage of their genes was compared using Kruskal–Wallis test, with Dunn’s multiple pairwise comparison in R (dunn.test – library^4^), with a significance of 0.05. Finally, to evaluate if there was a greater perturbation in DNA content close to replication origins after MMS treatment in TcTopo3α KO isolates, the copy number of DGF-1 genes close to replication origins described by Araujo and collaborators (de Araujo et al., 2020) was compared with the coverage of other DGF-1 genes, using Kruskal–Wallis/Dunn’s tests with a significance of 0.05 in R. The violin plot representing these results was generated in R, using the library GGplot2^5^. Visual representation of the gene-by-gene coverage variation values for all the five multigene families was generated in R, with raw counts or normalized by Z-score, using the Heatmap2 function. SNP and short insertion and deletion calls were performed with FreeBayes.v1.3.1, with ploidy ‘‘2,’’ a minimum of five reads supporting the alternate allele and a minimum mapping quality of 30 in the SNP position. The Venn diagram of the shared SNPs among the isolates was generated with the library ‘‘VennDiagram’’^6^ in R.

## Results

### Generation of TcTopo3α Knockout Cells

To investigate TcTopo3α function in *T. cruzi*, this gene was deleted in epimastigotes by disrupting both alleles with neomycin and hygromycin selectable resistance markers (Supplementary Figure 1A). The first allele was deleted using a pNEOΔTopo3α cassette, which carries neomycin-resistance gene. In order to confirm the insertion of deletion cassette in the correct locus, a PCR with the genomic DNA extracted from G418-resistant parasites and two different primer sets was performed (Supplementary Figure 1B). The PCR analysis showed that one allele of TcTopo3α gene was disrupted in parasites selected by neomycin-resistance gene. The amplification with PS2 confirmed that the full deletion cassette is present in genomic DNA of G418-resistant parasites. As expected, this primer set was also able to amplify the TcTopo3α gene in the intact allele. To delete the second allele, the single-knockout parasites were transfected with the pHYGROΔTopo3α cassette, which carries hygromycin B-resistance gene. To examine whether this cassette was inserted in the correct locus, another PCR using three different primer sets was performed (Supplementary Figure 1C). PCR analysis showed that neomycin and hygromycin resistance genes replaced both alleles of TcTopo3α. PCR assay, using a primer that hybridizes in a region inside the target gene, showed that TcTopo3α gene is only present in WT parasites.

**FIGURE 1:**
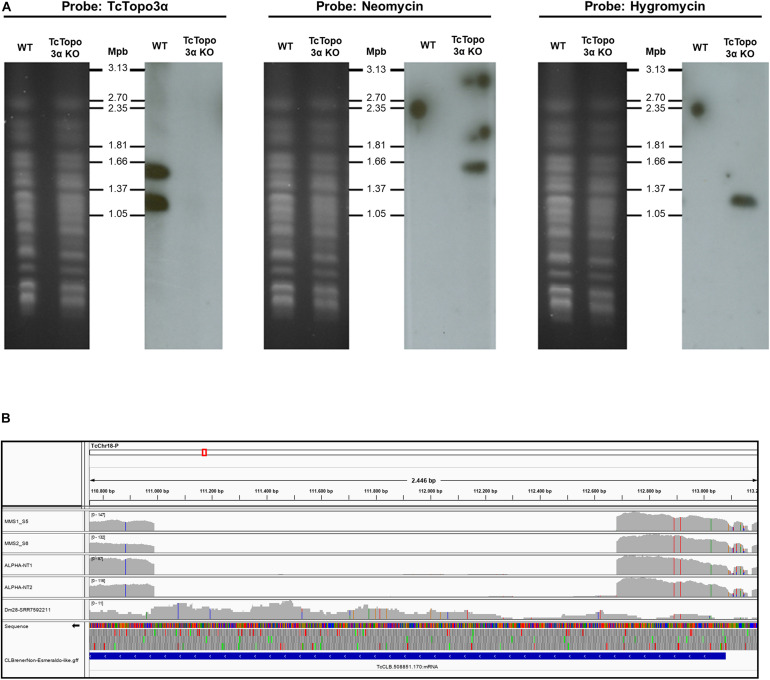
Confirmation of topoisomerase 3α gene knockout. **(A)** Southern blot analysis of WT and Topo3α KO parasites. The chromosomes of WT and knockout epimastigotes were separated by pulsed-field gel electrophoresis (PFGE) (panels on left), transferred to nylon membranes, and hybridized with radioactive probes for TcTopo3α, neomycin, or hygromycin resistance genes (panels on right). Chromosomes from yeast *Hansenula wingei* (1.05 to 3.13 Mbp) were used as molecular weight marker (Bio-Rad). It shows that TcTopo3α probe recognized bands only in WT parasites while neomycin and hygromycin probes hybridized with bands only in TcTopo3α KO parasites. It indicates that both TcTopo3α alleles were replaced by deletion cassettes in selected parasites. **(B)** TcTopo3α gene knockout confirmed by whole genome sequencing (WGS). In this integrative genomics viewer (IGV) image, the CL Brener Chr18 region that contains the TcCLB.508851.170 (TcTopo3α gene) is represented. The MM1, MM2, NT1, and NT2 boxes represents the read depth in these isolates, showing the absence of reads in the TcTopo3α gene central region. The Dm28 SRR7592211 box corresponds to the read depth in this isolate (obtained in NCBI), in which the TcTopo3α gene was not deleted. In these four boxes, gray bars correspond to the read depth in positions in which the reads were similar to the CL Brener reference genome, while blue, red, green, and orange bars correspond to a nucleotide change to, respectively, “C,” “T,” “A,” and “G.” The TcCLB.508851.170 gene region is represented by a blue box in the CL BrenerNon-Esmeraldo-like-gff box.

In addition, TcTopo3α gene knockout was confirmed by Southern blot using the radioactive probes that hybridize with neomycin-resistance, hygromycin-resistance, or TcTopo3α genes (Figure 1A). After the separation of chromosomes by PFGE, the TcTopo3α probe recognized two chromosomal bands only in WT parasites confirming that this gene was deleted in the mutant parasites. It has already been reported that there is a variation in size between homologous chromosomes in *T. cruzi* (Souza et al., 2011). Therefore, it is possible that the two bands observed in WT cells is due to a difference in size between the homologous chromosomes. In agreement, the neomycin probe hybridized with the bigger chromosome, while the hygromycin probe recognized the smaller chromosome in knockout cells. As expected, these probes did not hybridize with WT parasites. The TcTopo3α gene knockout was further confirmed by WGS, as shown by the absence of reads mapping into CL Brener TcCLB.508851.170 gene region (Figure 1B). Taken together, these results suggest that hygromycin B and neomycin resistance genes correctly replaced both TcTopo3α alleles, generating TcTopo3α KO parasites.

### Topoisomerase 3α Is Required for Amastigote Proliferation and Invasion but Does Not Impair Epimastigote Growth

**FIGURE 2:**
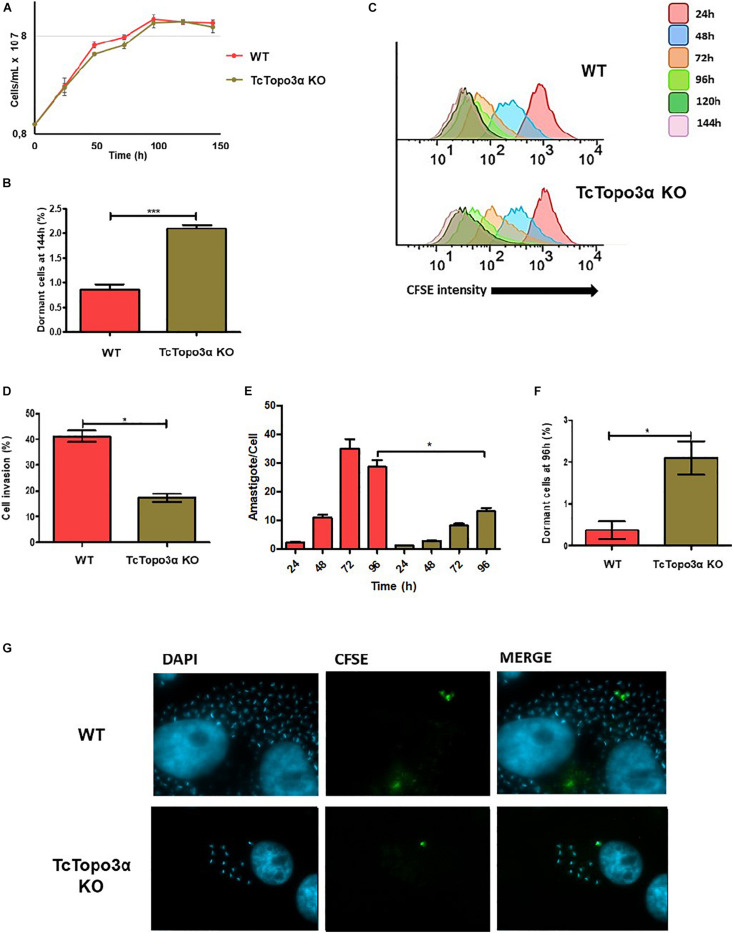
Proliferation behavior of TcTopo3α KO *T. cruzi* epimastigotes and amastigotes, and trypomastigote invasion profile. **(A)** Epimastigote cellular growth curves from WT and TcTopo3α KO. At 0 h, 1 × 10^7^ cells were treated with CFSE, and samples were counted every 24 h for 144 h. **(B)** Average of percentage of dormant cells in WT and TcTopo3α KO strains at 144 h was detected in flow cytometry histograms. **(C)** Flow cytometry histograms of epimastigote cultures from each strain from 24 to 144 h. CFSE intensity was assessed every 24 h until 144 h, and arrested cells were considered as those ones which exhibited similar CFSE intensity at 144 h when compared with the level of half median at 24 h. **(D)** Invasion rate of WT and TcTopo3α KO strains in LLC-MK2 cells. The graph shows the percentage of infected LLC-MK2 cells in a total of 250 cells that were analyzed (parasites labeled with anti-*T. cruzi* antibody were disregarded). **(E)** *T. cruzi* infection progression. The graph shows the number of intracellular parasites per infected cells. A total of 100 infected LLC-MK2 cells were analyzed at each time point at 96 h post-infection. **(F)** The graph shows the percentage of intracellular amastigotes in a dormant state 96 h post-infection. A total of 100 LLC-MK2 infected cells were analyzed. **(G)** Representative images of infected cultures, 96 h post-infection. Representative results of two distinct experiments in three technical triplicates. Asterisks indicate statistically significant differences among groups (*p*-value < 0.05).

Once in possession of a TcTopo3α KO parasite, the growth rate of its replicative forms, as well as the cellular invasion rate of its mammalian infective form was evaluated and compared with the WT strain. No statistically significant difference was observed in the growth curve when comparing epimastigote forms from knockout and WT strains (Figure 2A). However, when analyzing CFSE intensity upon 144 h of growth, TcTopo3α KO parasites showed a twofold increase in the number of cells presenting the maximum CFSE fluorescence when compared with WT strain (Figure 2B). This could imply an involvement of the TcTopo3α gene in duplication-arresting events in *T. cruzi*. In fact, when we analyzed CFSE intensity cytometer data measured over time, a widening of the peaks, especially 72 h after treatment, is observed prominently in the mutant cells, suggesting an intensification of asynchronous replication for these parasites (Figure 2C).

To assess the effect of TcTopo3α in the parasites’ ability to infect cells, the trypomastigote forms of WT and TcTopo3α KO parasites, obtained after three passages in LLC-MK2 cells, were used. Plated cells were infected with an MOI 50 for 1 h and fixed for immunofluorescence labeling. TcTopo3α KO parasites showed both a reduced invasion rate (Figure 2D), as well as a slower amastigote nest formation when compared with WT parasites (Figure 2E). Additionally, in order to follow dormant amastigotes (the ones that stopped duplication, retaining CFSE labeling), the trypomastigote forms were also labeled with CFSE before infection. Similar to that observed in epimastigotes, the rate of CFSE+ parasites was higher for TcTopo3α KO parasites, 2.1% versus less than 0.5% in the WT (Figure 2F). The CFSE+ amastigote forms are illustrated in Figure 2G.

### Topoisomerase 3α Is Important to Deal With DNA Double Strand Breaks Caused by Gamma Radiation

**FIGURE 3:**
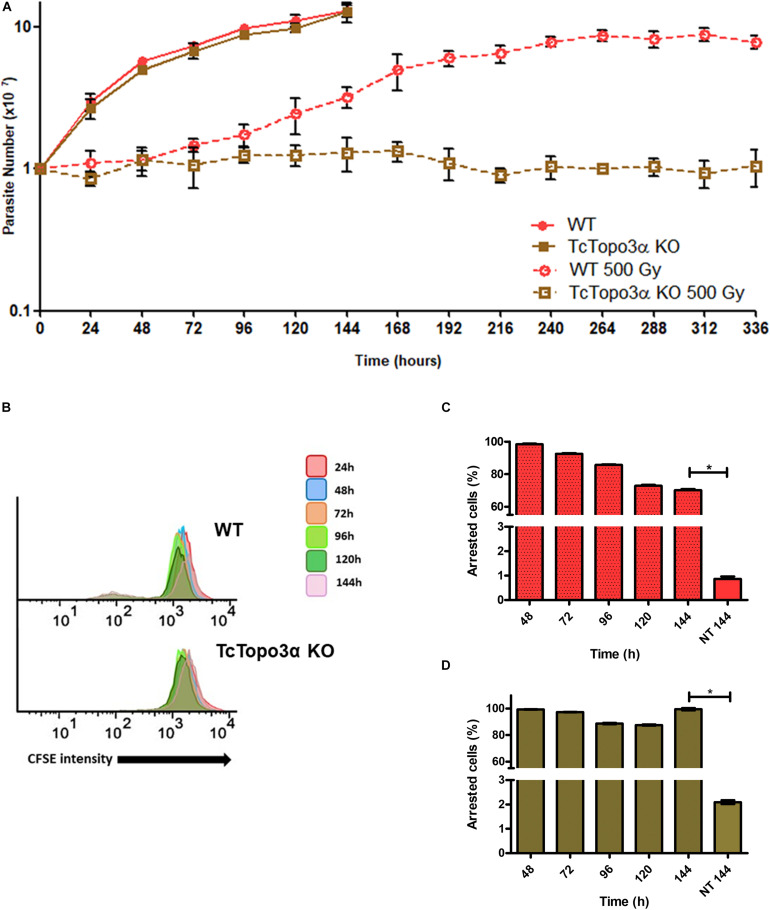
Growth curve and dormancy after gamma radiation. **(A)** Evaluation of the growth profile of WT and TcTopo3α KO *T. cruzi* epimastigotes parasites after exposure to 500 Gy of gamma radiation. The parasites were cultured in LIT medium at initial concentration of 1 × 10^7^ cells ml^– 1^ and counted in a cytometry chamber every 24 h. The experiment was performed in triplicate. **(B)** Flow cytometry histograms of CFSE fluorescence intensity decay over time from epimastigotes exposed to 500 Gy. Cells that have similar CFSE fluorescence intensity compared with the level of half median at time point of 24 h were considered as arrested. **(C,D)** The graph shows the percentage of WT **(C)** or TcTopo3α KO **(D)** arrested parasites determined by flow cytometry showing half of CFSE median intensity observed at 24 h of growth. The percentage of dormant parasites at 144 h – from a culture without exposure to HU (NT 144 h) is shown for the sake of comparison. Representative results of three distinct experiments in three technical triplicates. Asterisks indicate statistically significant differences among groups (*p*-value < 0.05).

To examine whether topoisomerase 3α acts in the homologous recombination in *T. cruzi*, WT and TcTopo3α KO cells were treated with gamma radiation. After irradiation with 500 Gy, both parasite lineages presented growth arrest for approximately 4 days, compared with parasites that were not exposed to gamma radiation (Figure 3A). After that, WT parasites were able to resume growth, whereas TcTopo3α KO cells did not resume growth during experiment analysis time (336 h). TcTopo3α KO growth arrest after irradiation was confirmed by the maintenance of CFSE labeling intensity over culture time (Figure 3B) as well as by the number of cells with maximum CFSE intensity (Figures 3C,D). While WT parasites showed a progressive reduction in CFSE labeling intensity upon 72 h of irradiation, TcTopo3α KO epimastigotes retained maximum CFSE florescence intensity up to 144 h of culture for almost all cells (Figures 3C,D).

### TcTopo3α KO Cells Are More Sensitive to Cisplatin and Hydroxyurea Treatments

**FIGURE 4:**
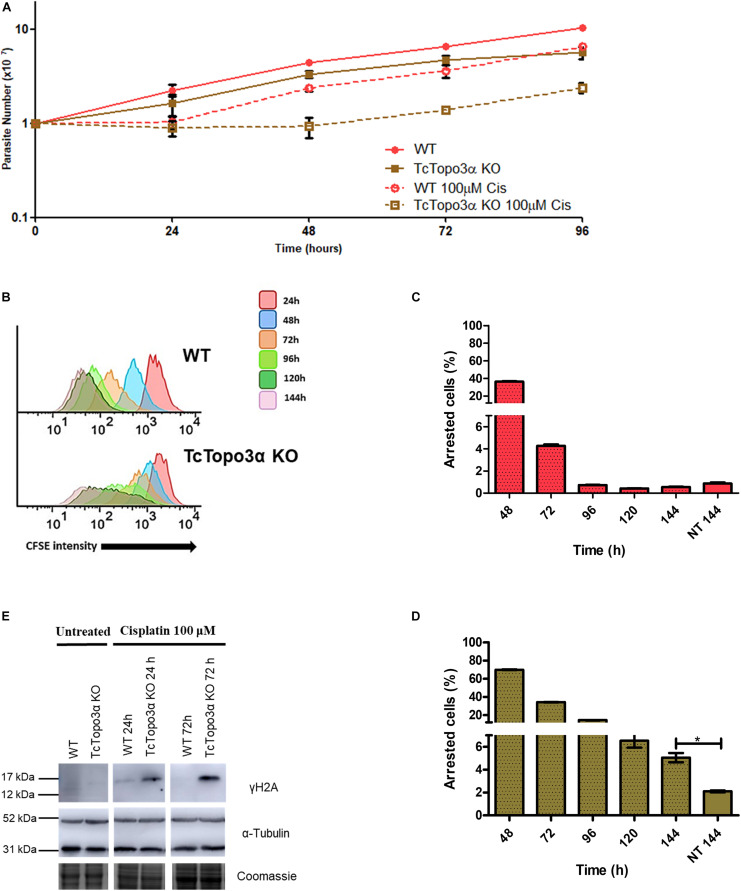
The role of topoisomerase 3α in the response to cisplatin treatment. **(A)** Growth curve after the treatment with 100 μM Cis for 1 h in PBS buffer. After the treatment, the cells were cultivated in fresh medium. The cells were counted every day using a cytometry chamber and vital dye erythrosine. It shows that TcTopo3α KO cells exposed to Cis delayed the resumption of growth in relation to WT cells. **(B)** Flow cytometry histograms of epimastigote cultures from each strain. CFSE fluorescence intensity was followed every 24 h until 144 h. Cells that exhibited similar CFSE fluorescence intensity at 144 h when compared with the level of half median at 24 h were considered as dormants. **(C,D)** Average of dormant cells’ percentages in WT **(C)** and TcTopo3α KO **(D)** cells at 144 h was detected in flow cytometry histograms. Representative results of three distinct experiments in three technical triplicates. Asterisks indicate statistically significant differences among groups (*p*-value < 0.05). **(E)** Western blot to measure the levels on DNA damage after the Cis treatment. The extracts were prepared at the indicated time points and were analyzed by Western blot with anti-γH2A antibody, a DSB marker. The anti-α-tubulin antibody was used as a loading control.

Due to the involvement of topoisomerase 3α in the DSB repair in *T. cruzi*, it was thought that this enzyme could also act during replication stress. To test this hypothesis, WT and TcTopo3α KO parasites were treated with cisplatin (Cis) or hydroxyurea (HU). Cisplatin interacts with N7 in purine residues generating intrastrand and interstrand crosslinks. These adducts cause DNA double helix distortions and block replication (Dasari and Tchounwou, 2014). HU inhibits ribonucleotide reductase reducing the deoxyribonucleotide pool available for DNA synthesis (Singh and Xu, 2016). In 24 h after Cis withdrawal, both WT and TcTopo3α KO parasites ceased growth in comparison with untreated cells (Figure 4A). Twenty-four hours later, WT cells were able to resume normal growth, while TcTopo3α KO cells resumed growth only 48 h later. In agreement with the growth curves, flow cytometry assay, performed 24 h after Cis treatment, showed cells arrested in the G2 phase of the cell cycle, for both parasite strains (Supplementary Figure 2). While WT parasites resumed normal cell cycle progression, a small part of TcTopo3α KO cells showed a sub-G1 DNA content. Additionally, the reduction in CFSE intensity after treatment was distinct in each cell. The peak enlargement observed for TcTopo3α KO cells, mainly after 96 h, reinforces their delayed growth resumption after Cis treatment. Also, these cells showed an intense asynchronous replication profile, since there were parasites with florescence intensity along the whole range analyzed (Figure 4B). After 144 h of Cis treatment, the number of cells unable to replicate since the initial time was higher in TcTopo3α KO cells when compared with their non-treated counterparts, which was not the case for treated and non-treated WT parasites (Figures 4C,D).

The role of topoisomerase 3α in the DNA damage repair during the replication stress induced by cisplatin was also assessed. In this sense, the level of γH2A, an initial modification during DNA damage response, was measured by Western blot after treatment. Anti-α-tubulin antibody was used as a loading control. For both WT and TcTopo3α KO parasites, the level of H2A phosphorylation increased 24 h after Cis treatment, indicating the presence of cisplatin-induced DNA damage (Figure 4E). The lesions in WT parasites were repaired until 72 h after Cis treatment. However, TcTopo3α KO cells remained with high levels of phosphorylated histone.

**FIGURE 5:**
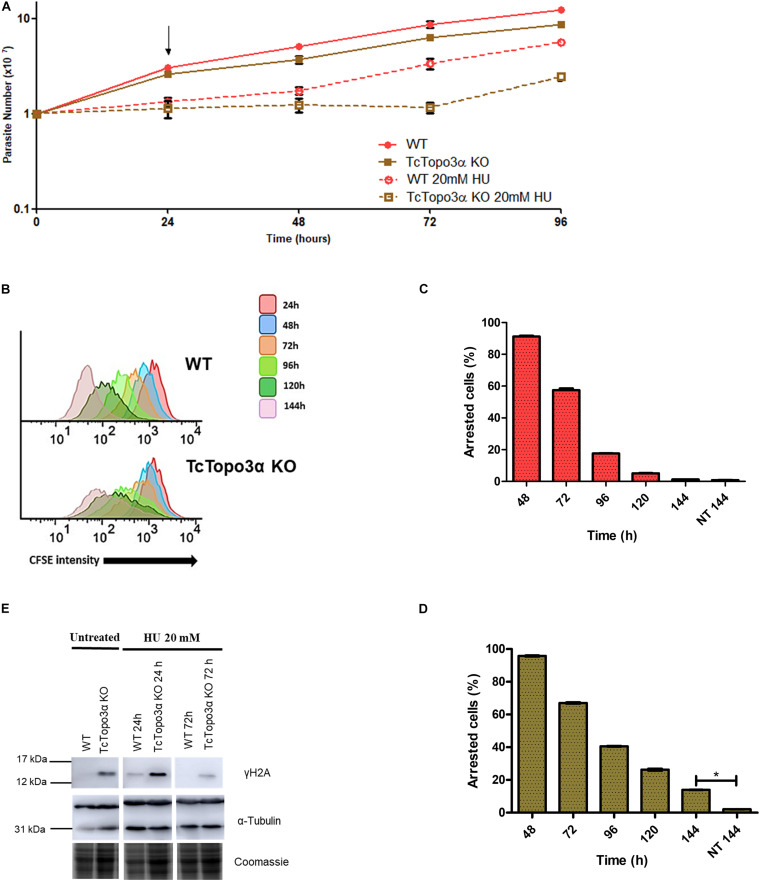
The role of topoisomerase 3α in the response to hydroxyurea (HU) treatment. **(A)** Growth curve after the treatment with 20 mM HU. The HU was kept in the LIT medium for 24 h. After that, the cells were washed and cultivated in a fresh medium. The cells were counted every day using a cytometry chamber and vital dye erythrosine. It shows that TcTopo3α KO cells exposed to HU delayed resumption of growth in relation to WT cells. The arrow indicates the time when HU was removed from the medium. **(B)** Flow cytometry histograms of CFSE fluorescence intensity decay over time from epimastigotes exposed to 20 mM HU. Cells that have similar CFSE fluorescence intensity compared to the level of half median at time point of 24 h were considered as arrested. **(C,D)** The graph shows the percentage of arrested parasites determined by flow cytometry showing half of CFSE median intensity observed at 24 h of growth. The percentage of dormant parasites at 144 h from a culture without exposure to HU (NT 144 h) is shown for the sake of comparison. Representative results of three distinct experiments in three technical triplicates. Asterisks indicate statistically significant differences among groups (*p*-value < 0.05). **(E)** Western blot to measure the levels on DNA damage after the treatment with 20 mM HU. The extracts were prepared at the indicated time points and were analyzed by Western blot with anti-γH2A antibody. The anti-α-tubulin antibody was used as a loading control. Treatment with HU for 24 h increased levels of γH2A in both parasites. The WT cells were able to repair their lesions at the time of 72 h, which did not happen with Topo3α KO parasites. In the absence of HU, only TcTopo3α KO cells exhibited bands for the phosphorylated histone.

Similar to cisplatin, HU treatment halted growth for 24 h for both WT and TcTopo3α KO parasite lineages when compared with their non-treated counterparts (Figure 5A). After HU removal from the culture medium, WT parasites were able to recover growth. In contrast, TcTopo3α KO cells remained stalled for about 2 days before resuming growth. In conformity with the growth curves, flow cytometry analysis showed that 24 h of HU treatment was able of synchronize the parasites in the G1/S phase of the cell cycle (Supplementary Figure 3). Later, WT cells were able to resume normal cell cycle progression, while a slight amount of TcTopo3α KO cells exhibited a sub-G1 DNA content, similar to Cis treatment effect on the replicative progression. The assessment of TcTopo3α KO CFSE florescence intensity labeling after treatment revealed parasites stalled in every generation formed after labeling (Figure 5B). High CFSE-labeled, non-replicating/dormant cell numbers were also higher after 144 h of HU treatment for TcTopo3α KO cells when compared with HU-treated WT parasites (Figures 5C,D).

The Western blot analysis showed that after treatment with HU for 24 h, both WT and TcTopo3α KO cells had γH2A bands, suggesting HU induced DNA damage. Drug removal from culture medium allowed the WT parasites to repair their lesions until 72 h. The same was not observed for TcTopo3α KO parasites, which could not repair completely their DNA damage up to this time, similar to cisplatin (Figure 5E).

### The Absence of Topoisomerase 3α Impairs the Growth Resumption and DNA Repair After Methyl Methanesulfonate Treatment

**FIGURE 6:**
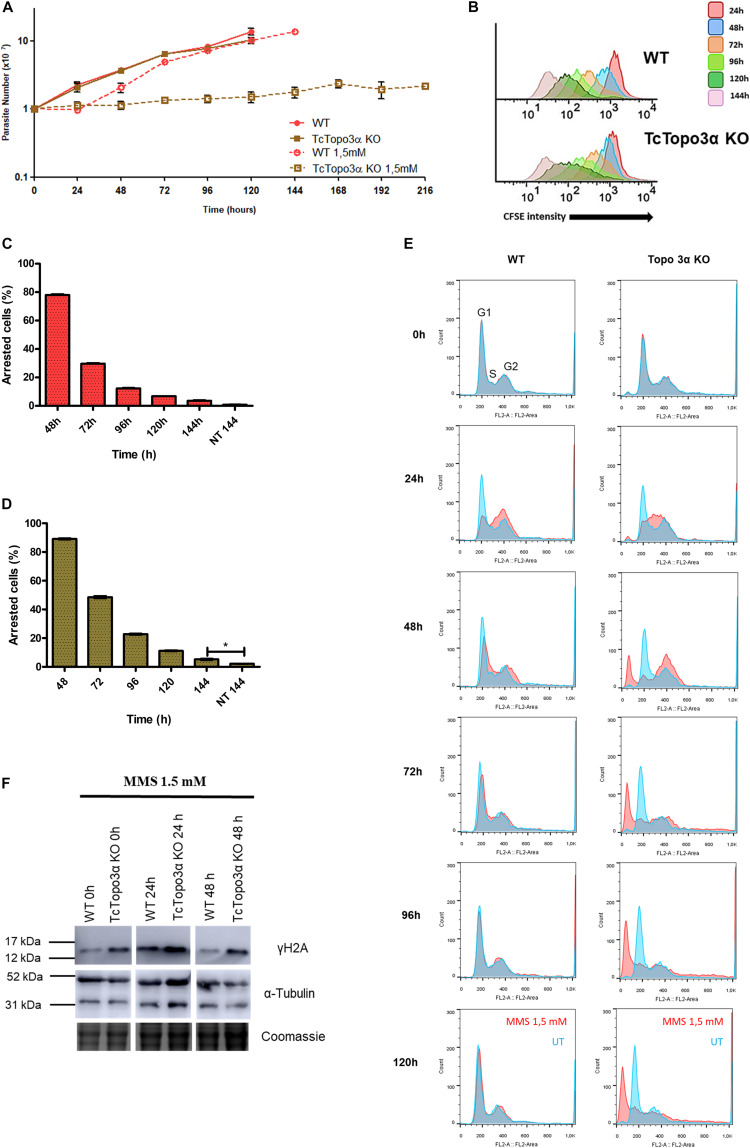
The role of topoisomerase 3α in the response to methyl methanesulfonate (MMS) treatment. **(A)** Growth curve after the treatment with 1.5 mM MMS for 1 h in PBS buffer. After the treatment, the cells were cultivated in fresh medium. The cells were counted every day using a cytometry chamber and vital dye erythrosine. It shows that Topo3α KO cells exposed to MMS are not able to resume growth. **(B)** Flow cytometry histograms of CFSE fluorescence intensity decay over time from epimastigotes exposed to 1.5 mM MMS. Cells that have similar CFSE fluorescence intensity compared with the level of half median at time point of 24 h were considered as arrested. **(C,D)** The graph shows the percentage of arrested parasites determined by flow cytometry showing half of CFSE median intensity observed at 24 h of growth. The percentage of dormant parasites at 144 h from a culture without exposure to MMS (NT 144 h) is shown for the sake of comparison. Asterisks indicate statistically significant differences among groups (*p*-value < 0.05). **(E)** Histogram of cell cycle progression after MMS treatment. The cells were analyzed by flow cytometry after being labeled with propidium iodide. In all the histograms, the blue curves represent the untreated cells, and the red ones refer to the cells exposed to the drug. It shows that, for both parasites tested, MMS promoted accumulation of cells in the S-phase at the time of 24 h. WT parasites were able to resume normal cell cycle progression at later times. However, the same was not observed in Topo3α KO cells, which accumulated in the sub-G1. Representative results of three distinct experiments in three technical triplicates. **(F)** MMS-induced DNA damage. Both parasites increased the γH2A levels 24 h after MMS treatment. In the time of 48 h, WT cells were able to repair DSBs, while TcTopo3α KO parasites did not. Even without treatment, the absence of topoisomerase 3α increased the γH2A levels. The extracts were prepared at the indicated time points and were analyzed by western blot with anti-γH2A antibody, a DSB marker. The anti-α-tubulin antibody was used as a loading control.

Wild-type and TcTopo3α KO parasites were also treated with MMS, which methylates single or double-strand DNA. Some alkylated bases are able to stall replicative polymerases (Wyatt and Pittman, 2006). The growth curve showed that both parasite populations presented a growth impairment for 24 h after MMS treatment (Figure 6A). In the following 24 h, WT parasites were able to recover growth unlike mutant parasites. Even after 216 h, TcTopo3α KO cells did not restore growth. Nonetheless, CFSE intensity progressively reduced over time, for both WT and TcTopo3α KO parasites, indicating that cellular division events are occurring despite maintenance of the cell number in culture (Figure 6B). These results strongly suggest the occurrence of cell death in TcTopo3α KO population at a rate comparable to the replication rate. In fact, 144 h after MMS treatment, the number of cells that did not undergo a single division event were only 5% of TcTopo3α KO total parasites, yet the number is higher than in non-treated ones. The same statistical difference was not observed in WT parasites at 144 h of culture (Figures 6C,D).

Flow cytometry analysis showed that MMS changed the progression of the *T. cruzi* cell cycle when compared with untreated cells (Figure 6E). WT and TcTopo3α KO cells accumulated in the S/G2 phase 24 h after MMS treatment. Later, WT parasites resumed normal cell cycle progression. On the other hand, TcTopo3α KO parasites increased the number of cells in the G2 phase at 48 h, which would suggest an ability of these parasites in resuming the cell cycle. However, these cells were not able to resume normal cell cycle progression, accumulating in the sub-G1.

In order to examine the ability of TcTopo3α KO cells to repair MMS-induced DNA damage, a Western blot was performed. At 24 h after the MMS treatment, WT and TcTopo3α KO parasites increased the γH2A levels (Figure 6F). At 48 h, TcTopo3α KO cells had a more intense γH2A band than WT cells, which suggests that the absence of topoisomerase 3α impairs the DNA damage to be fully repaired.

Aiming to better characterize the impact of MMS treatment in TcTopo3α KO lineages, WGS was used to evaluate genomic modifications at chromosomal, segmental, and gene levels. The description of the number of reads, genome coverage, SNPs, indels, and NCBI SRA ID can be seen in the Supplementary Table 2. A total of 680,181 SNP positions were identified when all the samples were compared with the CL Brener reference genome. From those, 462,101 (∼68%) were shared among all isolates (Supplementary Figure 11). There was no significant change in the pattern of chromosomal duplication/deletion among MMS treated (MM1 and MM2) and untreated (NT1 and NT2) isolates. The extra copies of chromosome 19 and 31, and the potential loss of copies from chromosome 28 predate MMS treatment, as they were observed both in treated and non-treated parasites (Supplementary Figure 4).

**FIGURE 7:**
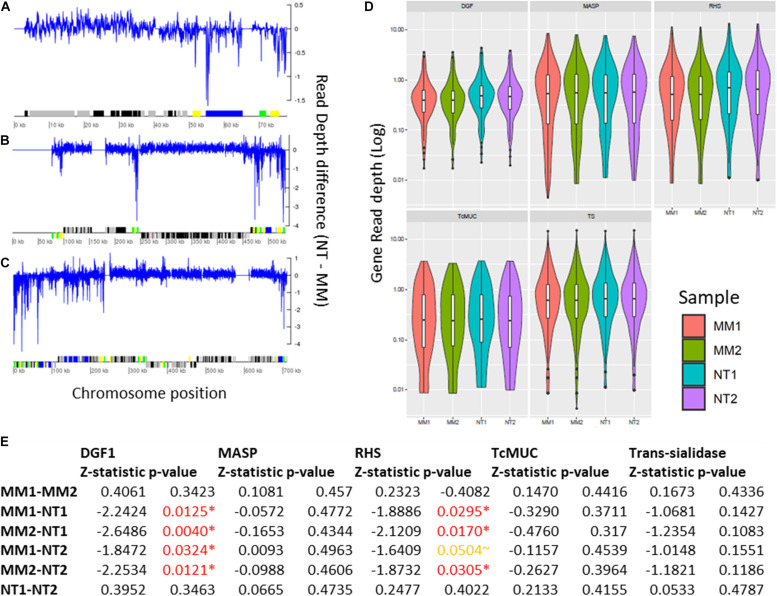
Impact of MMS treatment in segmental and gene duplication/loss in TcTopo3α KO cells. Segmental duplications in Chromosomes 1 **(A)**, 12 **(B)**, and 22 **(C)**. In this image, the blue line corresponds to the difference between the read depth of NT (1–2) and MM (1–2) across the whole chromosome sequence, where values above and below zero correspond, respectively, to increase copies in MM (1–2) and NT (1–2). Below, protein-coding genes are depicted as rectangles drawn in proportion to their length, and their coding strand is indicated by their position above (top strand) or below (bottom strand) the central line. Colored boxes represent: DGF-1 (blue); GP63 (Pink); MASP (brown); RHS (green); and Trans-sialidase (yellow); hypothetical genes (black); or other genes (gray). Gaps are represented by gene-less regions with no read coverage. **(D)** Violin plot representing the gene copy number of DGF-1, MASP, RHS, TcMUC, and Trans-Sialidase genes. The *Y*-axis represents the distribution of gene coverages, in log scale. MM1 (red), MM2 (green), NT1 (blue), and NT2 (purple). **(E)** Z-statistic and *p*-values of Dunn’s test between the comparisons of DGF-1, MASP, RHS, TcMUC, and trans-sialidase gene coverages. *P*-values < 0.05 are highlighted in red, and *p*-values∼0.05 are highlighted in orange. There was a significant reduction in the DGF-1 and RHS copy numbers in MMS-treated TcTopo3α KO isolates.

Segmental duplications/deletions, estimated as the difference in read depth between MMS treated and untreated TcTopo3α KO cells, at each position of the 41 *T. cruzi* chromosomes was also evaluated. Several potential small-scale segmental insertion/deletions were observed, where the MMS treatment-associated DNA loss appears to be more intense in multigene family’s regions, especially closer to DGF-1, RHS, and Trans-sialidase genes, as seen in Chr1, 12, and 22 (Figures 7A–C and Supplementary Figures 5–7). To further evaluate the MMS impact on multigene families, copy number in TcTopo3α KO cells, the gene-by-gene coverage from each *T. cruzi* multigene family DGF-1, MASP, RHS, TcMUC, and trans-sialidase was evaluated and compared between treated and non-treated parasites. As seen in the segmental duplication/deletion analysis, there was a statistically significant decrease in the overall coverage of DGF-1 and RHS genes (*p* < 0.05), an almost significant decrease in trans-sialidase (*p*∼0.14) (Figures 7D,E) and no statistically significant decrease in MASP and TcMUC, in MMS-treated TcTopo3α KO parasites when compared with MMS-treated WT. This lower copy number in each DGF-1, RHS, and to a lesser extent in trans-sialidase does not appear to be caused by the loss of specific genes. Instead, it appears to be widespread in the majority of the genes from these three families (Supplementary Figure 8). The DNA loss also do not appear to be associated with regions of DNA-replication origins, as no significant difference in coverage of DGF-1 genes that were near or far from replication origins was observed (Supplementary Figure 9).

### Telomeric Content Might Be Affected by Methyl Methanesulfonate Treatment in TcTopo3α Knockout Cells

Once the MMS treatment generated sub-G1 cells (Figure 6E), with less DNA content, we evaluate whether DNA losses at chromosomes telomeric regions occurred. Both TcTopo3α KO and WT parasites were treated with MMS for 72 h with DNA samples extracted every 24 h. The telomere content was analyzed in Southern blot experiments, using the telomeric repeat TTAGGG as probe (Supplementary Figure 10A). The hybridized probe revealed a distinct pattern of telomeric sequence distribution between DNA samples from WT and TcTopo3α KO cells. TcTopo3α KO DNA had higher bands than WT DNA, with higher intensity upon ImageJ analysis (Supplementary Figure 10B). In addition, after 72 h of MMS treatment, TcTopo3α KO DNA exhibited less intense stained DNA by the probe hybridization than WT DNA (Supplementary Figure 10B middle panel). This reduced intensity, incompatible with the DNA load control gel and not observed in WT cells (Supplementary Figure 10B left panel), could suggest a telomere loss after MMS treatment in TcTopo3α KO cells.

### Topoisomerase 3α Is Necessary to Solve Hydroxyurea- and Methyl Methanesulfonate-Induced Replication Stress

**FIGURE 8:**
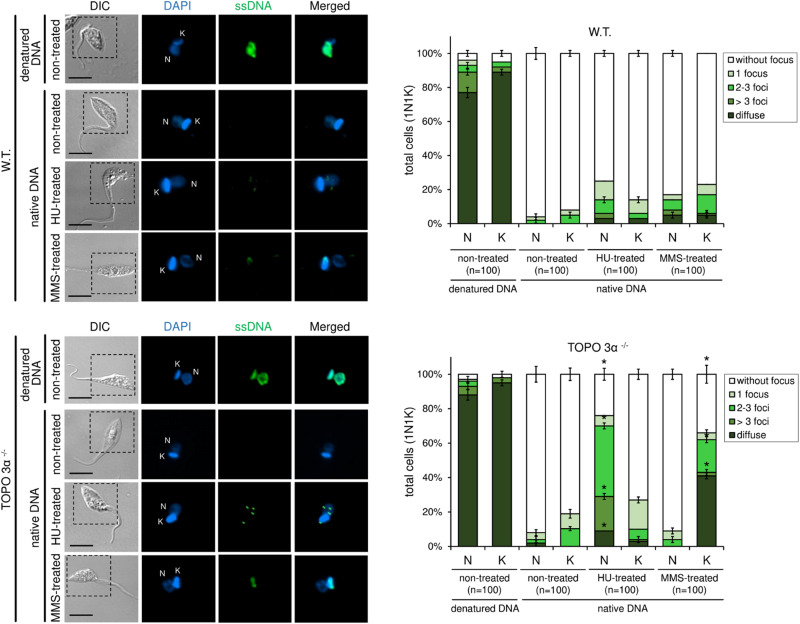
5-bromo-2′-deoxyuridine (BrdU) native detection assay reveals that TcTopo3α KO parasites are more sensitive to genotoxic stress caused by HU or MMS. WT parasites naturally show very low levels of single-stranded DNA (ssDNA) foci either in the nucleus (N) or kinetoplast (k). After HU treatment, the number of ssDNA foci (green) per cell, especially in the nucleus, increases significantly in TcTopo3α KO parasites relative to WT. However, after MMS treatment, the pattern and the number of ssDNA foci per cell increased in kinetoplast but not in the nucleus, showing a diffuse pattern in some cells and a dotted pattern in others. The bar graphs show the measurement of ssDNA foci distribution pattern according to each organelle (N and k) per cell. As a positive control, each lineage was treated with HCl to denature DNA and consequently expose the ssDNA. Black bars = 10 μm. Error bars indicate SD. The differences observed were statistically significant relative to control using the Student’s *t*-test (*p < 0.05); *n* = 100 for each condition analyzed.

To further assess the role of topoisomerase 3α in the replication stress response, a BrdU native detection assay was carried out. In this assay, it is possible to measure the levels of ssDNA foci, which is considered a replication stress marker. In normal culture conditions, both WT and TcTopo3α KO parasites naturally showed very low levels of ssDNA foci either in the nucleus (N) or kinetoplast (K) (Figure 8). After HU treatment, the number of ssDNA foci per cell, especially in the nucleus, increased significantly in knockout parasites relative to the WT. However, after MMS treatment, the pattern and the number of ssDNA foci per cell increased in kinetoplast but not in the nucleus, showing a diffuse pattern in some cells and a dotted pattern in others (Figure 8).

### TcTopo3α Knockout Cells Are Not Sensitive to ATR Signaling

**FIGURE 9:**
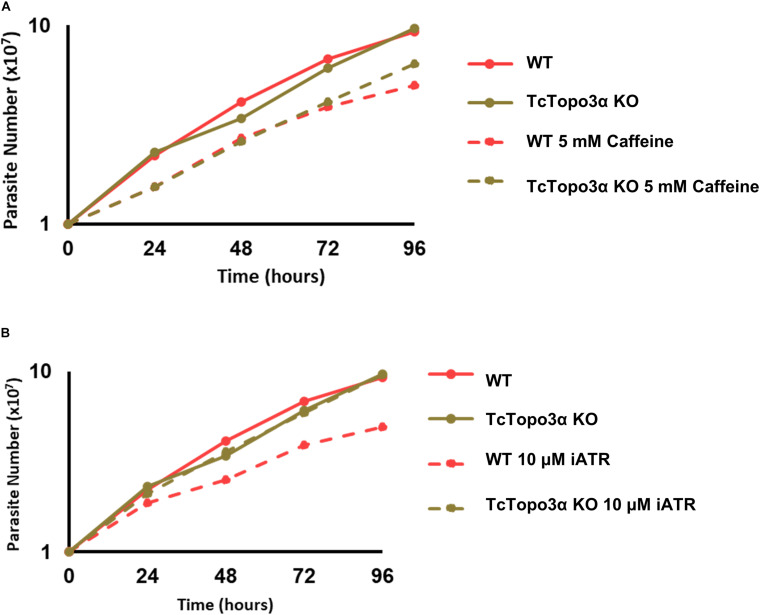
The effect of ataxia telangiectasia mutated (ATM)/ataxia telangiectasia and Rad3-related (ATR) inhibition in TcTopo3α KO parasites. **(A)** Growth curve after the treatment with 5 mM caffeine. The caffeine was kept in the LIT medium during all experiment time analysis (144 h). **(B)** Growth curve after the treatment with 10 μM of ATR inhibitor (VE-821). The ATR inhibitor was kept in the LIT medium during all experiment time analysis (144 h). The cells were counted every day using a cytometry chamber and vital dye erythrosine. Representative results of three distinct experiments are shown.

It is known that gamma irradiation and its DBS generation activates ATM kinase signaling, while MMS and its intense alkylation on DNA induce formation of ssDNA and ATR kinase signaling. Both pathways act in stalling replication and cellular cycle. Once we observed the discrepancy in CFSE decay between gamma radiation and MMS treatments, but without altering cellular concentration in culture, we checked the response of the TcTopo3α KO and WT parasites to ATM/ATR kinase signaling. To that end, the parasites were treated with caffeine, an unspecific inhibitor of both ATM and ATR kinases, and VE-821, a specific ATR inhibitor, in normal growth conditions. Interestingly, in the presence of caffeine, both cellular types were affected with impaired growth (Figure 9A). However, only WT cells were affected by the ATR inhibitor, showing reduced growth rate (Figure 9B). These data suggest that TcTopo3α KO parasites are no longer sensitive to ATR kinase signaling.

## Discussion

For most Chagasic chronic patients, there are no physical signs or clinical evidence of injury to any organ during their entire lives (Malik et al., 2015). However, about 20 to 30 years after the initial infection, asymptomatic patients may develop organ complications (Pérez-Molina et al., 2015). It is unclear what happens to the parasite during all these years of asymptomatic infection and what factors allow the disease to reactivate. A recent study has shown that after infection, some *T. cruzi* amastigotes spontaneously become dormant (Sánchez-Valdéz et al., 2018). Such cells are able to resume cell proliferation and reestablish infection, even after treatment with trypanocidal drugs. The mechanisms involved in controlling dormancy are not completely known yet, but it was demonstrated in the involvement of homologous recombination in this process. Partial deletion of the TcRad51 gene caused a reduction in the rate of natural dormancy in the *T. cruzi* CL Brener strain, and that the rate of TcRad51 transcription between different strains is related to the rate of natural dormancy (Resende et al., 2020). In this work, we showed that the complete deletion of TcTopo3α in *T. cruzi* Dm28c strain caused a reverse effect to what is observed for TcRad51, with an increase in dormancy in TcTopo3α KO parasites, in both replicative forms (Figure 2). Together, these data not only reinforce the influence of homologous recombination on the dormancy processes in *T. cruzi* but also suggest that the homologous recombination intermediates, not solved by TcTopo3α, could be acting as a signal for dormancy. Interestingly, these modifications in the dormancy rate in both TcTopo3α and TcRad51 mutants were not enough to change the epimastigote growth in culture (Figure 2A), even though CFSE labeling has shown the asynchronous nature of replication in these parasites (Figure 2B). On the other hand, again as observed for parasites with partial deletion of TcRad51 (Gomes Passos Silva et al., 2018), cell invasion and amastigote multiplication rates were reduced in TcTopo3α KO parasites (Figure 2; Resende et al., 2020). The fact that the depletion of different genes involved in homologous recombination (TcRad51 and TcTopo3α) have a similar effect in reducing invasion and replication in the mammalian host cell, despite their antagonistic effect in dormancy induction, suggests differences in recombination process for each form of *T. cruzi* life cycle, whether replicative or not (Gomes Passos Silva et al., 2018).

In contrast to WT cells, TcTopo3α KO parasites did not reestablish normal growth rates after exposure to 500 Gy of gamma radiation (Figure 3A), which suggests an involvement of this enzyme in the DSB repair in *T. cruzi*, as previously reported for other organisms (Bizard and Hickson, 2014). Corroborating this hypothesis, the CFSE labeling intensity of TcTopo3α KO gamma radiated parasites did not decrease, even after 144 h of culture (Figures 3B,D). This suggests that an intense rate of DSB caused in DNA leads to activation of ATM kinase, and consequentially to cell cycle arrest and activation of homologous recombination repair. It is possible that accumulation of unsolved Holliday junctions in TcTopo3α KO parasites induces a generalized state of dormancy, perhaps due to the continuous ATM kinase signaling. Since stalled replication forks can collapse and also generates DSBs, the role of topoisomerase 3α during replication stress in *T. cruzi* was investigated.

Treatment with 1.5 mM MMS halted WT and TcTopo3α KO parasite growth for 24 h (Figure 6A). Consistent with this growth arrest, there was an increase in the level of H2A phosphorylation in both parasites 24 h after treatment (Figure 6F). To promote lesion repair, the cells arrested the cell cycle in the S/G2 phase (Figure 6E). After 24 h, WT parasites were able to repair the DNA damage caused by MMS and resume cellular growth and cell cycle progression. In contrast, TcTopo3α KO parasites still had considerable levels of γH2A 48 h after MMS treatment (Figure 6F). Curiously, cell duplication analysis via CFSE labeling showed that, different from what was observed for gamma radiation treatment, in which CFSE fluorescence remains maximum in 100% of the cells analyzed until 144 h of culture (Figure 3B), in TcTopo3α KO MMS-treated parasites, only 5% of the cells have shown maximum CFSE intensity 144 h after treatment, strongly suggesting the occurrence of cellular division events (Figure 6B). Nonetheless, the number of parasites remained the same, even after a long period, suggesting that duplication events are balanced by cell death events (Figure 6A). In accordance, these parasites were able to resume cell cycle progression, as there was an increase in the number of cells in the G2 phase 48 h after treatment. However, TcTopo3α KO parasites accumulated in the sub-G1 over time (Figure 6E).

Since these sub-G1 TcTopo3α KO MMS-treated parasites remained alive even with reduced DNA content, we attempted to characterize their genome. A Southern blot carried out with probes for repetitive telomeric regions suggested telomere loss after 72 h in MMS (Supplementary Figure 10). It is well known that Holliday junctions in DNA move in both directions of the DNA strand by Ruv-like enzymes to complete repair of this structure (Shinagawa and Iwasaki, 1996). Therefore, assuming TcTopo3αKO parasites’ inability to resolve Holliday junction structures, it is plausible to assume that once not repaired, Holliday junctions are taken by Ruv to face regions such as telomeric or secondary structures. The latter can lead to DSB in DNA by locking the fork replication in cell mitosis events. Since the mechanisms of response to cell stress are disabled in these parasites, these breaks may be leading to the loss of total DNA in the cells, which would be responsible for the increase in population at sub-G1.

To further characterize the impact of MMS in *T. cruzi* at the genome-sequence level, WGS reads of MMS-treated (5 days treatment) and untreated (control) TcTopo3α KO parasites were generated. CCNV analysis showed no differential alteration in chromosomal somies between MMS-treated and untreated parasite populations, suggesting that MMS perturbation was unable to cause chromosomal copy instability in *T. cruzi*. Even tough ploidy variations are widespread among *T. cruzi* DTUs (Reis-Cunha et al., 2015), the rate and mechanisms enrolled in chromosomal expansion/loss in this parasite are still unknown. However, while chromosomal copy gains (trisomy or tetrasomy) are common in *T. cruzi*, chromosomal loss (monosomy or chromosomal absence) are rare (Reis-Cunha et al., 2018). As DNA alkylating agents such as MMS usually result in mispairing and replication blocks, it could potentially result in chromosomal copy loss, which could be lethal to the parasite. This could contribute to the population growth arrest observed in MMS-treated TcTopo3α KO parasites.

In contrast to what was seen at the chromosomal level, MMS treatment appears to cause short-scale DNA loss in TcTopo3α KO isolates, especially in multigene family’s clusters enriched in DGF-1, RHS, and to a lesser extent, trans-sialidases (Figure 7). There are two non-excluding possibilities to explain these findings. First, DGF-1 and RHS-rich regions could be directly or indirectly affected by MMS treatment in TcTopo3α KO cells. As MMS modifies guanine to 7-methylguanine (Beranek, 1990; Lundin et al., 2005), and many DGF-1 genes are GC enriched (de Araujo et al., 2020) and have a very GC-biased codon usage (Kawashita et al., 2009), this higher density of guanines could be a hotspot of mutation in the MMS treatment. Since GC-rich regions have a propensity to form strong DNA secondary structures (Zhao et al., 2017; Mao et al., 2018) and, in general, are associated with increased recombination rates (Kiktev et al., 2018), mutations in this region could potentially not be resolved in the absence of TcTopo3α topoisomerase. The absence of a preferential loss of DGF-1 near replication origins (Supplementary Figure 9), and the sub-telomeric localization of some DGF-1 and ∼30% or RHSs (Bernardo et al., 2020) suggests that the DNA loss caused by MMS treatment was not associated with the start of DNA replication. In fact, structural instabilities in the DNA molecule that could not be resolved in the absence of the TcTopo3α could be “pushed” away from the replication origin by the DNA replication fork, until they find a highly structured region, such as DGF-1′s G-quadruplexes. The latter could result in a stalled replication fork and, consequentially, DNA loss. This hypothesis is in accordance with the potential telomere shortening, observed in Southern blot assays (Supplementary Figure 10). The second possibility would be that perturbation and gene loss in DGF-1 and RHS genes could be non-lethal or less detrimental to the parasite than mutations in other genomic regions or multigene families. Hence, what was observed is the “mild” effect of MMS in TcTopo3α KO cultures, while more severe alterations were lethal. Hence, DGF-1 and RHS loss could be a “survivorship bias” in MMS-treated TcTopo3α KO parasites.

Methyl methanesulfonate methylated bases are usually repaired by BER. However, in trypanosomatids, no specific DNA glycosylase capable of removing N-methyl purines has so far been identified (Charret et al., 2012). Recently, it was shown that MMS-induced lesions are repaired by NER and homologous recombination in *T. brucei* (Vieira-da-Rocha et al., 2018). In *T. cruzi*, Rad51 single knockout parasites were sensitive to treatment with 1.5 mM MMS. Moreover, this treatment was able to increase γH2A levels in TcRad51+/− parasites, suggesting that TcRad51 is necessary to cope with the replication stress generated by this alkylating agent (Gomes Passos Silva et al., 2018). Therefore, it is possible to suggest that the absence of TcTopo3α impairs the completion of homologous recombination during replication stress caused by MMS. In humans, it was observed that the persistent presence of homologous recombination repair intermediates in the G2 phase increases the signaling of ATR kinase and promotes the activation of cellular senescence via p21 (Feringa et al., 2018). In *T. cruzi*, however, the duplication was corroborated with the decrease in CFSE labeling intensity after treatment with MMS (Figure 6). In fact, our experiments using both an ATR-specific inhibitor and a non-specific ATM/ATR inhibitor (caffeine) revealed that TcTopo3α KO cells are not sensitive to the ATR signaling (Figure 9). Our hypothesis is that, in order to survive in the absence of TcTopo3α, there is a selective pressure for those parasites not responsive to the ATR kinase signaling, since in this condition ATR could cause cell cycle arrest due to the constant accumulation of ssDNA (Figure 8). Thus, it is possible to speculate that the absence of topoisomerase 3α during this type of replication stress triggers a state of cellular senescence in *T. cruzi*. Further studies need to be performed to confirm this hypothesis.

Cisplatin treatment also halted WT and TcTopo3α KO growth for 24 h (Figure 4A). In accordance with our growth curve results, evaluation of cell cycle progression showed an accumulation of treated cells in the G2 phase of the cell cycle 24 h after treatment (Supplementary Figure 2), as previously observed for mammalian cells (Sorenson et al., 1990; Wagner and Karnitz, 2009; Lützkendorf et al., 2017). Again, measurement of DNA lesions by Western blot using anti-γH2A antibody confirmed cisplatin-induced DNA damage 24 h after treatment (Figure 4E). WT parasites repaired their lesions during cell cycle arrest, which allowed them to resume cell cycle progression and growth at the later times. On the other hand, 72 h after cisplatin treatment, TcTopo3α KO parasites still had lesions in the DNA. This persistent DNA damage delayed the resumption of growth in these parasites and promoted a small accumulation of these cells in the sub-G1. Since we have previously shown that there is no difference between TcRad51+/− and WT parasite survival 48 h after cisplatin treatment (Gomes Passos Silva et al., 2018), we here suggest that topoisomerase 3α is required to solve the replication stress caused by cisplatin in *T. cruzi* by a pathway other than homologous recombination.

Hydroxyurea treatment for 24 h was able to synchronize both WT and TcTopo3α KO parasites in the G1/S phase of the cell cycle (Supplementary Figure 3), as standardized for other *T. cruzi* strains (Galanti et al., 1994), as well as DNA damage (Figure 5E). HU removal from the culture medium allowed the recovery of growth of WT parasites (Figure 5A), as well as the recovery of normal cell cycle progression (Supplementary Figure 3). Also, at 72 h, no bands for γH2A were observed, indicating that all lesions caused by HU were repaired. In the case of TcTopo3α KO cells, they were able to repair part of their lesions, but still had a weak γH2A detection at 72 h of culture (Figure 5E). Upon HU removal, even if the level of γH2A is close to that found in untreated cells, the fact that these parasites were able to continue the progression of the cell cycle, but with a slight accumulation of cells in the sub-G1, and a delayed cell growth recovery, compared with WT, suggests the involvement of this protein in the resolution of lesions caused by HU (Supplementary Figure 3 and Figure 5A). In humans, repair of DSBs generated by prolonged treatment with HU requires the Rad51 protein (Petermann et al., 2010). However, in *T. cruzi*, there is no difference between TcRad51+/− and WT parasites growth profile after HU treatment. In addition, the level of phosphorylated H2A after treatment with 20 mM HU in these two cells lineages is very similar, suggesting that in *T. cruzi*, HU-induced lesions are not repaired by homologous recombination (Gomes Passos Silva et al., 2018). Once again, it is possible to suggest that topoisomerase 3α is necessary to deal with HU-induced replication stress, although it seems that homologous recombination is not involved in this process. The replication stress caused by both Cis and HU treatments was enough to intensify asynchronous replication and dormancy (Figures 4, 5), suggesting once more the influence of TcTopo3α unsolved substrates in inducing duplication stalling.

Taken together, the results obtained here indicate that TcTopo3α is required during homologous recombination repair, as previously described for other organisms (Bizard and Hickson, 2020). Moreover, topoisomerase 3α also acts during the replication stress caused by cisplatin, HU, and MMS in *T. cruzi*. It is possible to note that not all responses to replication stress are dependent on homologous recombination, which may explain the variation in sensitivity of Topo3α KO parasites to the different genotoxic agents tested. Replication stress induced by MMS can be solved via homologous recombination pathway. However, it is possible that topoisomerase 3α also acts in other pathways to allow the recovery of the replication forks after the replication stress caused by HU and cisplatin. The mechanisms recruited after these treatments need to be elucidated. Furthermore, it was also shown that the absence of TcTopo3α and, therefore, the inability to solve the intermediates of the recombination processes, is an important signal for dormancy or asynchronous replication in epimastigote and amastigote forms. Thus, understanding the role of topoisomerase 3α in *T. cruzi* may contribute to further elucidate the biology of this trypanosomatid and the mechanisms of its successful infection in the vertebrate host.

## Data Availability Statement

The data presented in the study are deposited in the NCBI SRA repository, accession numbers: SRR13754247, SRR13754250, SRR13754249, and SRR13754248.

## Author Contributions

HC-S, BR, LA, JR-C, GF, SF, and CM conceptualized the study. HC-S, BR, AU, WP, MS, SV, AM, ET, LT, ME, LA, JR-C, GF, SF, and CM performed the formal analysis. HC-S, BR, AU, WP, MS, SV, ME, LA, JR-C, GF, SF, and CR performed the investigations. The original draft was written by HC-S, BR, WP, and MS. The study was written by ME, LA, JR-C, GF, SF, and CM. Funding was secured by AM, SP, ET, LT, LA, GF, SF, and CR, while resource sourcing was done by AM, SP, ET, LT, ME, LA, JR-C, GF, SF, and CR. LA, JR-C, GF, SF, and CR did the methodology. All authors contributed to the article and approved the submitted version

## Conflict of Interest

The authors declare that the research was conducted in the absence of any commercial or financial relationships that could be construed as a potential conflict of interest.

## Footnotes

1 https://www.bioinformatics.babraham.ac.uk/projects/fastqc/

2 https://www.r-project.org/

3 https://rdrr.io/cran/genoPlotR/man/genoPlotR-package.html

4 https://cran.r-project.org/web/packages/dunn.test/index.html

5 https://cran.r-project.org/web/packages/ggplot2/index.html

6 https://cran.r-project.org/web/packages/VennDiagram/index.html

## Supplementary Material

Supplementary Figure 1 Generation of TcTopo3α KO parasites. **(A)** Schematic representation of the deletion cassettes pNEOΔTopo3α or pHYGROΔTopo3α (top panel), TcTopo3α locus in WT parasites (middle panel) and the locus generated in knockout parasites after transfection (bottom panel). Arrows indicate the primer sets used in the PCR to confirm the correct insertion of deletion cassettes. **(B)** Ethidium bromide stained gel showing PCR products generated with primer sets PS1 (lane 1) and PS2 (lane 2) using genomic DNA of TcTopo3α KO epimastigotes. It indicates that pNEOΔTopo3α cassette was insert correctly in the TcTopo3α locus of one allele. **(C)** Ethidium bromide stained gel showing PCR products generated with primer sets PS1 (lane 1), PS3 (lane 2), and PS4 (lanes 3 and 4) using genomic DNA of WT or TcTopo3α KO epimastigotes. It indicates that pNEOΔTopo3α and pHYGROΔTopo3α cassettes were insert correctly in the TcTopo3α locus of both alleles generating knockout parasites.

Supplementary Figure 2 Cell cycle progression after Cis treatment. Representative histogram and quantification of cell cycle progression analysis after the treatment with or 100 μM Cis. The cells were analyzed by flow cytometry after being labeled with propidium iodide. In all the histograms, the blue curves represent the untreated cells and the red ones refer to the cells exposed to the drug. It shows that, for both parasites tested, both doses of Cis promoted accumulation of cells in the G2 phase in the time of 24 h. Later, WT parasites were able to resume normal cell cycle progression while there was a slight accumulation of TcTopo3α KO cells in the sub-G1 phase.

Supplementary Figure 3 Cell cycle progression after HU treatment. Representative histogram and quantification of cell cycle progression analysis after the treatment with 20 mM HU. The cells were analyzed by flow cytometry after being labeled with propidium iodide. In all the histograms, the blue curves represent the untreated cells and the red ones refer to the cells exposed to the drug. It shows that, for both parasites tested, different doses of HU synchronized the cells in the G1/S phase in the time of 24 h. Later, WT parasites were able to resume normal cell cycle progression while there was a slight accumulation of TcTopo3α KO cells in the sub-G1 phase.

Supplementary Figure 4 Somy variation among MMS treated and untreated TcTopo3α KO *T. cruzi* isolates. In each image, the *Y*-axis corresponds to the median coverage of all genes in a chromosome normalized by genome coverage, represented in a boxplot, where the median value corresponds to the chromosome-predicted somy. Each bar on the x-axis represents a *T. cruzi* chromosome. A median value of ∼1 means that the chromosomally estimated somy was similar to the genome ploidy. No significant alteration was observed in treated and untreated isolates.

Supplementary Figures 5–7 Impact of MMS treatment in segmental duplication/loss in TcTopo3α KO cells. In this image, the blue line corresponds to the difference between the read depth of NT (1-2) and MM (1-2) across the whole chromosome sequence, where values above and below zero correspond, respectively, to increase copies in MM (1-2) and NT (1-2). Below, protein-coding genes are depicted as rectangles drawn in proportion to their length, and their coding strand is indicated by their position above (top strand) or below (bottom strand) the central line. Colored boxes represent: DGF-1 (blue); GP63 (Pink); MASP (brown); RHS (green) and Trans-sialidase (yellow); hypothetical genes (black) or other genes (gray). Gaps are represented by gene-less regions with no read coverage.

Supplementary Figure 8 Heatmap of gene-by-gene copy number variation in *T. cruzi* multigene families, in MMS treated MM (1-2) and untreated NT (1-2) TcTopo3α KO isolates. In this image, each line corresponds to an *T. cruzi* isolate, and each column corresponds to a gene. The copy number of each gene is represented in a scale from blue to red, denoting low and high counts, respectively. Columns (representing genes) and rows (representing samples) were clustered by UPGMA, based on the Manhattan distance of raw gene coverages. For each multigene family, two heatmaps were generated, one containing the read depth normalized by genome coverage (Raw-Counts), and one with the values normalized by Z-score by column. There was a significant reduction in counts on DGF-1 and RHS gene families, and a mild reduction in Trans-sialidases in MM1 and MM2, when compared with NT1 and NT2.

Supplementary Figure 9 Violin plot representing the gene copy number of DGF-1 genes near replication origins. **(A)** The *Y* axis represent the distribution of gene coverages, in log scale. MMS-treated DGF-1 non-origin (MMSNO – red), MMS-treated DGF-1 origin (MMSO – green), Non-Treated non-origin (NTNO – blue), and Non-Treated origin (NTO – purple). **(B)** Z-statistic and *p*-values of Dunn’s test. *P*-values < 0.05 are highlighted in red, and *p*-values ∼0.05 are highlighted in orange. There was no statistically supported difference in coverages of DGF-1 close or distant from replication origins (MMSNO-MMSO and NTNO-NTO).

Supplementary Figure 10 Telomere length analysis by Southern blot. **(A)** Purified probe containing telomeric sequences repeat (TTAGGG)∼50 was generated by pTEL plasmid digestion with HindIII restriction enzyme. **(B)** Southern blot analysis of telomeric sequence-containing fragments generated by digestion of genomic DNA with restriction enzymes CviQI, HpaII, AluI, and HhaI; genomic DNA from WT and TcTopo3α KO cells was analyzed. Ethidium bromide-stained agarose gel showing the digestion products that were further analyzed by Southern blot. Right panel represents quantification of signal intensities for telomere hybridization using ImageJ software. Signals were plotted against DNA size (Kb).

Supplementary Figure 11 Venn diagram of the SNPs in the MMS1, MMS2, NT1, and NT2 isolates. Venn diagram depicting the sharing of the SNP positions in the four *T. cruzi* evaluated isolates.
