# Supplementary figures and images for: DNA Topoisomerase 3α Is Involved in Homologous Recombination Repair and Replication Stress Response in Trypanosoma cruzi
Source: Front Cell Dev Biol. 2021 May 13;9:633195w. doi: 10.3389/fcell.2021.633195 (PMC8155511; doi:10.3389/fcell.2021.633195)

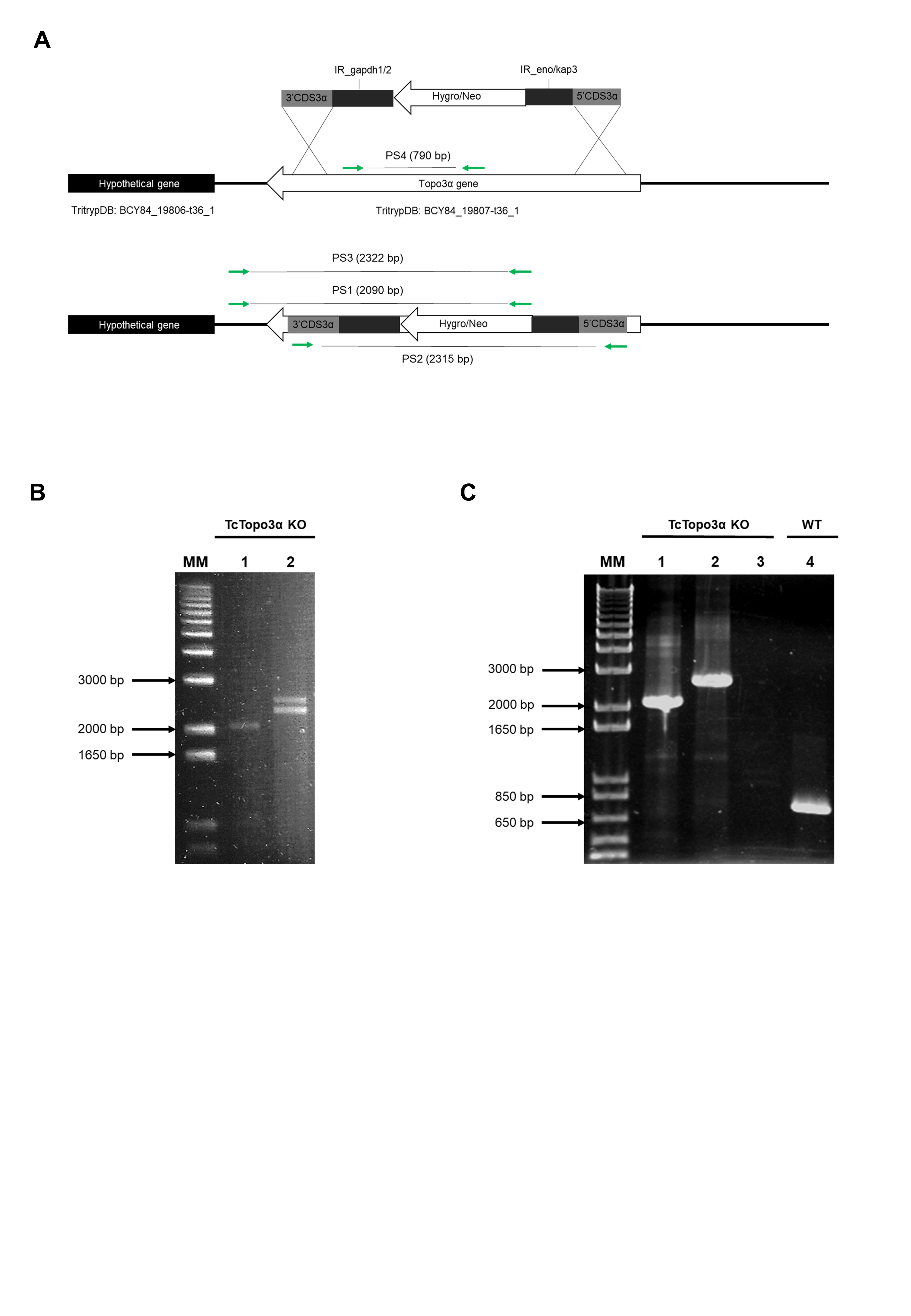

Supplement: Supplementary Figure 1 — Generation of TcTopo3α KO parasites. (A) Schematic representation of the deletion cassettes pNEOΔTopo3α or pHYGROΔTopo3α (top panel), TcTopo3α locus in WT parasites (middle panel) and the locus generated in knockout parasites after transfection (bottom panel). Arrows indicate the primer sets used in the PCR to confirm the correct insertion of deletion cassettes. (B) Ethidium bromide stained gel showing PCR products generated with primer sets PS1 (lane 1) and PS2 (lane 2) using genomic DNA of TcTopo3α KO epimastigotes. It indicates that pNEOΔTopo3α cassette was insert correctly in the TcTopo3α locus of one allele. (C) Ethidium bromide stained gel showing PCR products generated with primer sets PS1 (lane 1), PS3 (lane 2), and PS4 (lanes 3 and 4) using genomic DNA of WT or TcTopo3α KO epimastigotes. It indicates that pNEOΔTopo3α and pHYGROΔTopo3α cassettes were insert correctly in the TcTopo3α locus of both alleles generating knockout parasites. [file Image_1.TIF]

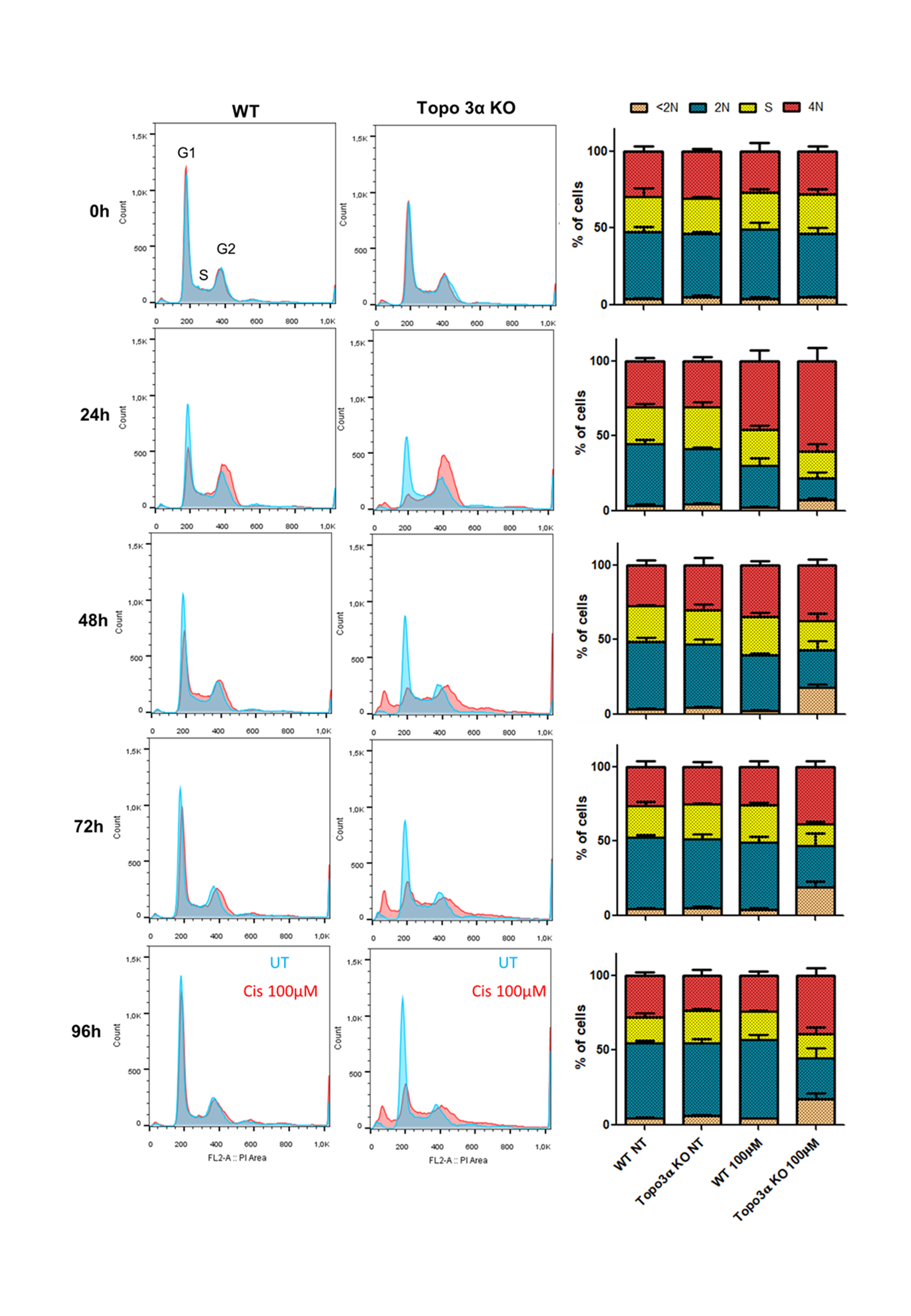

Supplement: Supplementary Figure 2 — Cell cycle progression after Cis treatment. Representative histogram and quantification of cell cycle progression analysis after the treatment with or 100 μM Cis. The cells were analyzed by flow cytometry after being labeled with propidium iodide. In all the histograms, the blue curves represent the untreated cells and the red ones refer to the cells exposed to the drug. It shows that, for both parasites tested, both doses of Cis promoted accumulation of cells in the G2 phase in the time of 24 h. Later, WT parasites were able to resume normal cell cycle progression while there was a slight accumulation of TcTopo3α KO cells in the sub-G1 phase. [file Image_2.TIF]

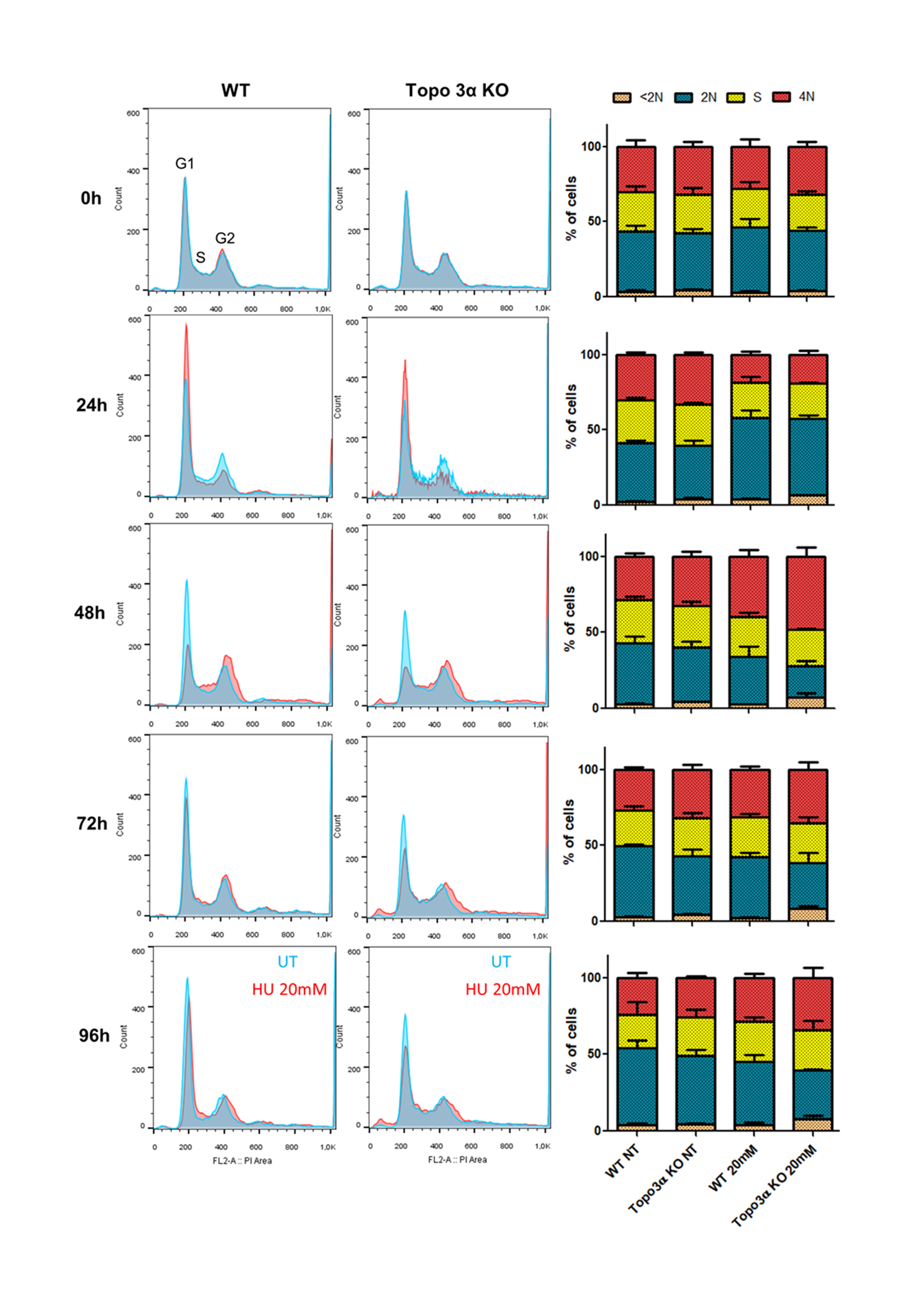

Supplement: Supplementary Figure 3 — Cell cycle progression after HU treatment. Representative histogram and quantification of cell cycle progression analysis after the treatment with 20 mM HU. The cells were analyzed by flow cytometry after being labeled with propidium iodide. In all the histograms, the blue curves represent the untreated cells and the red ones refer to the cells exposed to the drug. It shows that, for both parasites tested, different doses of HU synchronized the cells in the G1/S phase in the time of 24 h. Later, WT parasites were able to resume normal cell cycle progression while there was a slight accumulation of TcTopo3α KO cells in the sub-G1 phase. [file Image_3.TIF]

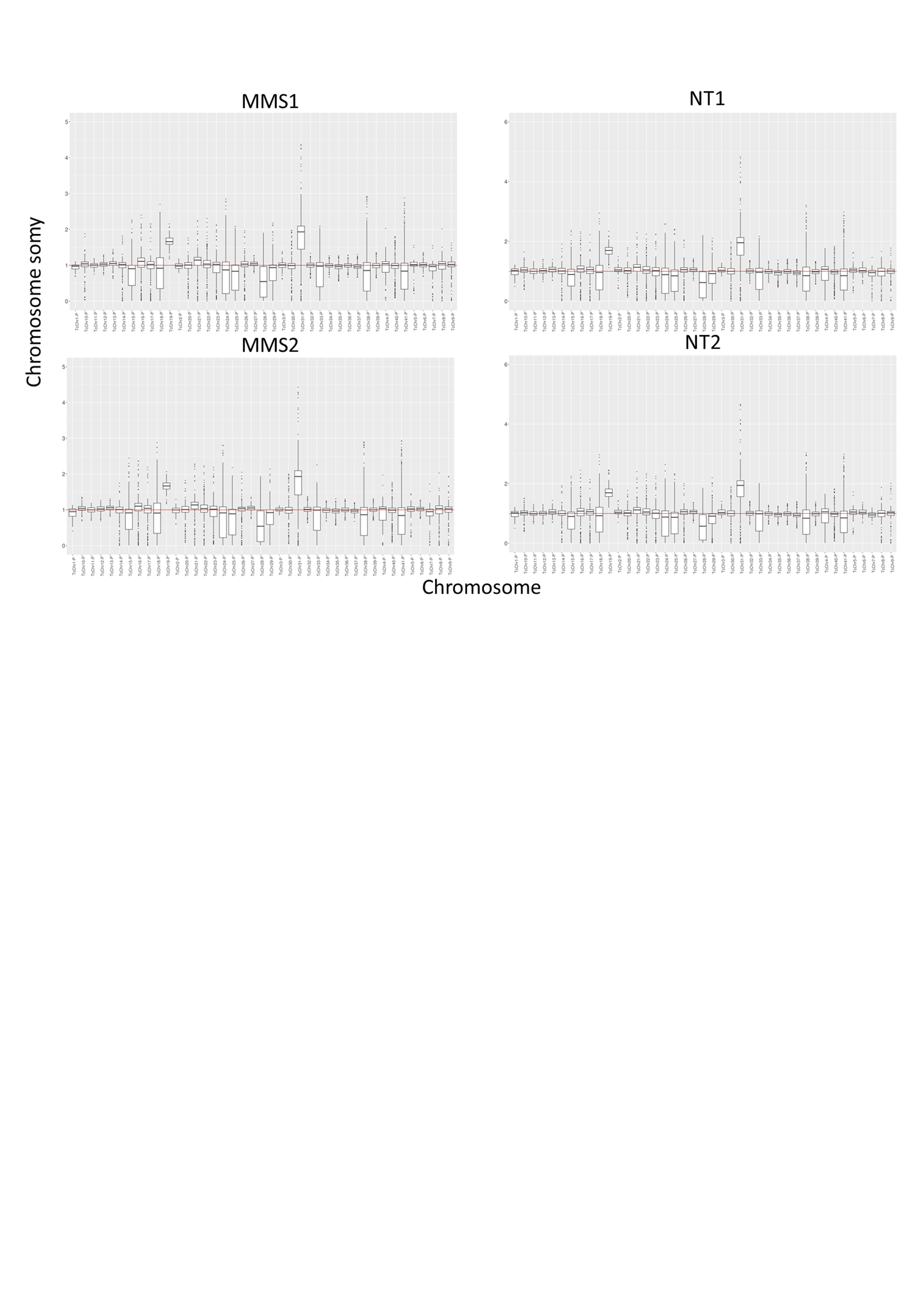

Supplement: Supplementary Figure 4 — Somy variation among MMS treated and untreated TcTopo3α KO T. cruzi isolates. In each image, the Y-axis corresponds to the median coverage of all genes in a chromosome normalized by genome coverage, represented in a boxplot, where the median value corresponds to the chromosome-predicted somy. Each bar on the x-axis represents a T. cruzi chromosome. A median value of ∼1 means that the chromosomally estimated somy was similar to the genome ploidy. No significant alteration was observed in treated and untreated isolates. [file Image_4.TIF]

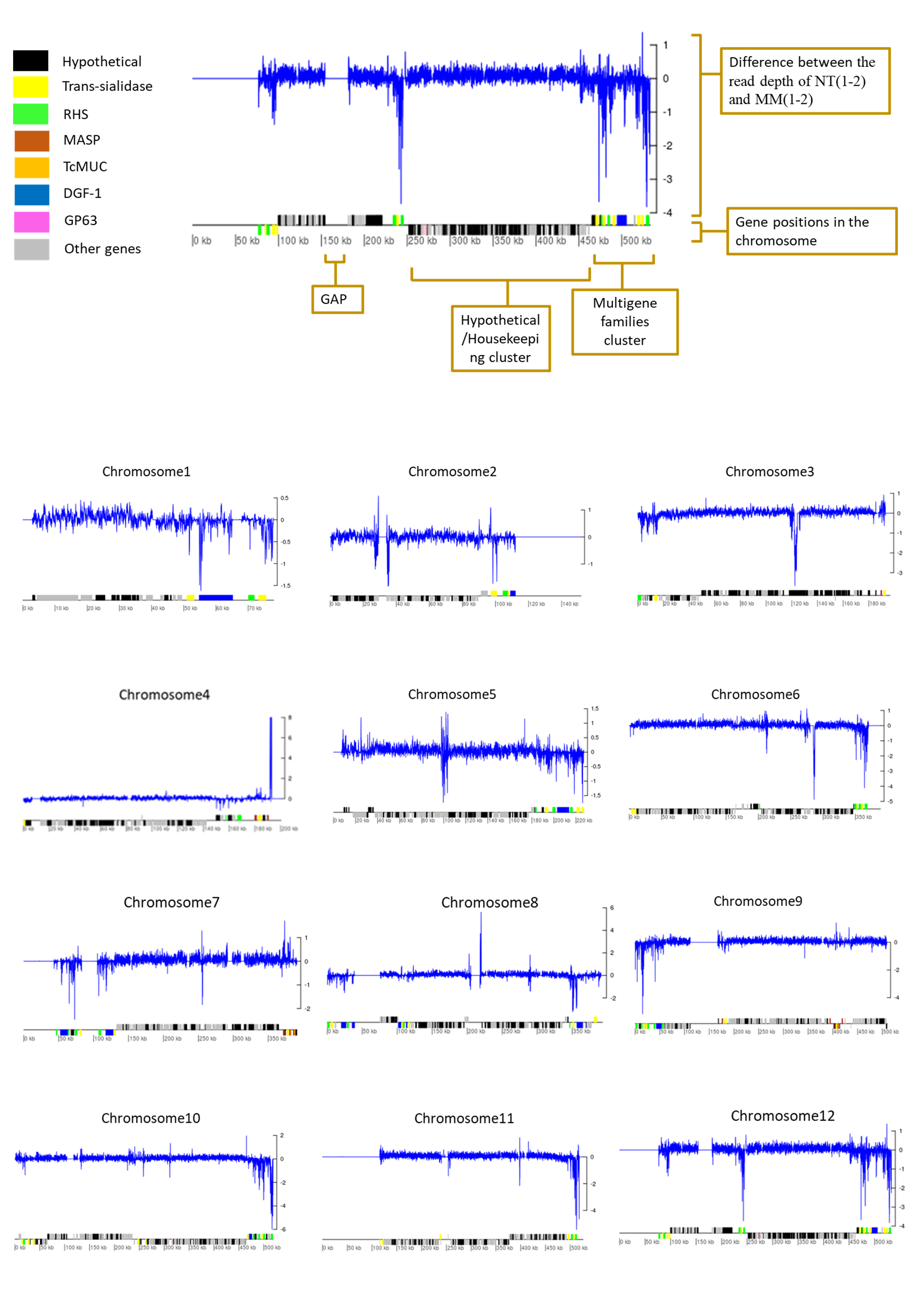

Supplement: Supplementary Figures 5–7 — Impact of MMS treatment in segmental duplication/loss in TcTopo3α KO cells. In this image, the blue line corresponds to the difference between the read depth of NT (1-2) and MM (1-2) across the whole chromosome sequence, where values above and below zero correspond, respectively, to increase copies in MM (1-2) and NT (1-2). Below, protein-coding genes are depicted as rectangles drawn in proportion to their length, and their coding strand is indicated by their position above (top strand) or below (bottom strand) the central line. Colored boxes represent: DGF-1 (blue); GP63 (Pink); MASP (brown); RHS (green) and Trans-sialidase (yellow); hypothetical genes (black) or other genes (gray). Gaps are represented by gene-less regions with no read coverage. [file Image_5.TIF]

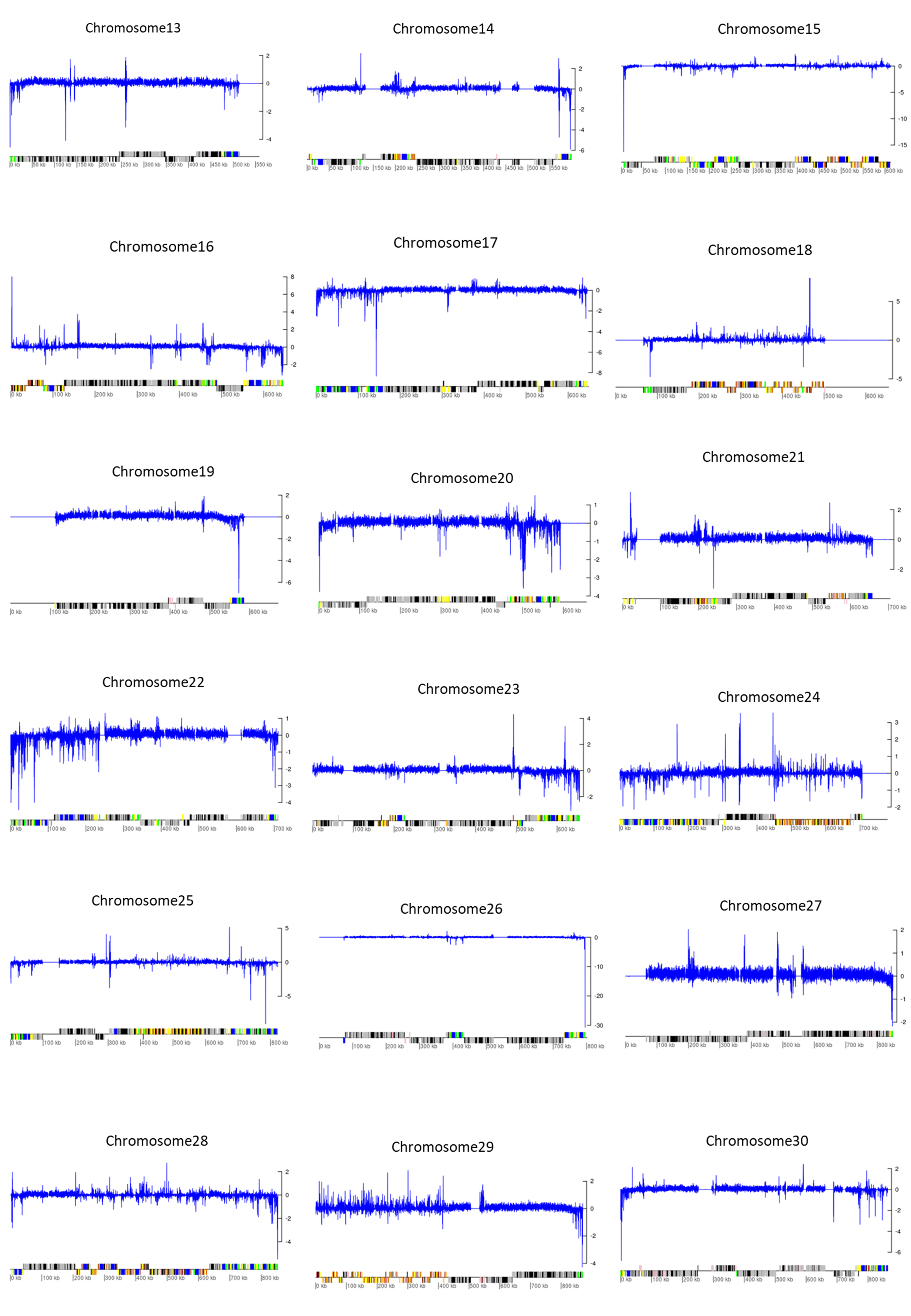

Supplement: Supplementary file 6 [file Image_6.TIF]

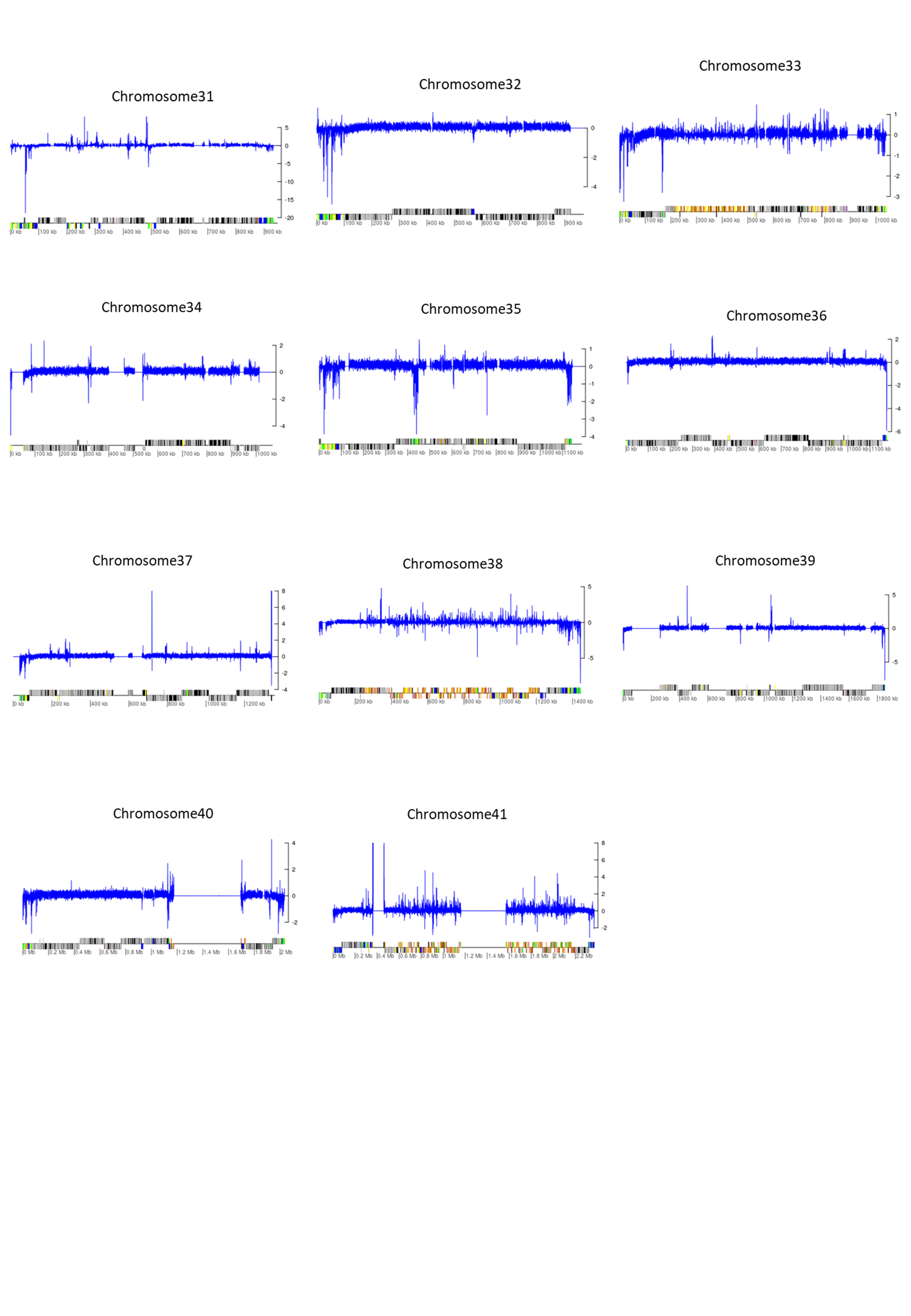

Supplement: Supplementary file 7 [file Image_7.TIF]

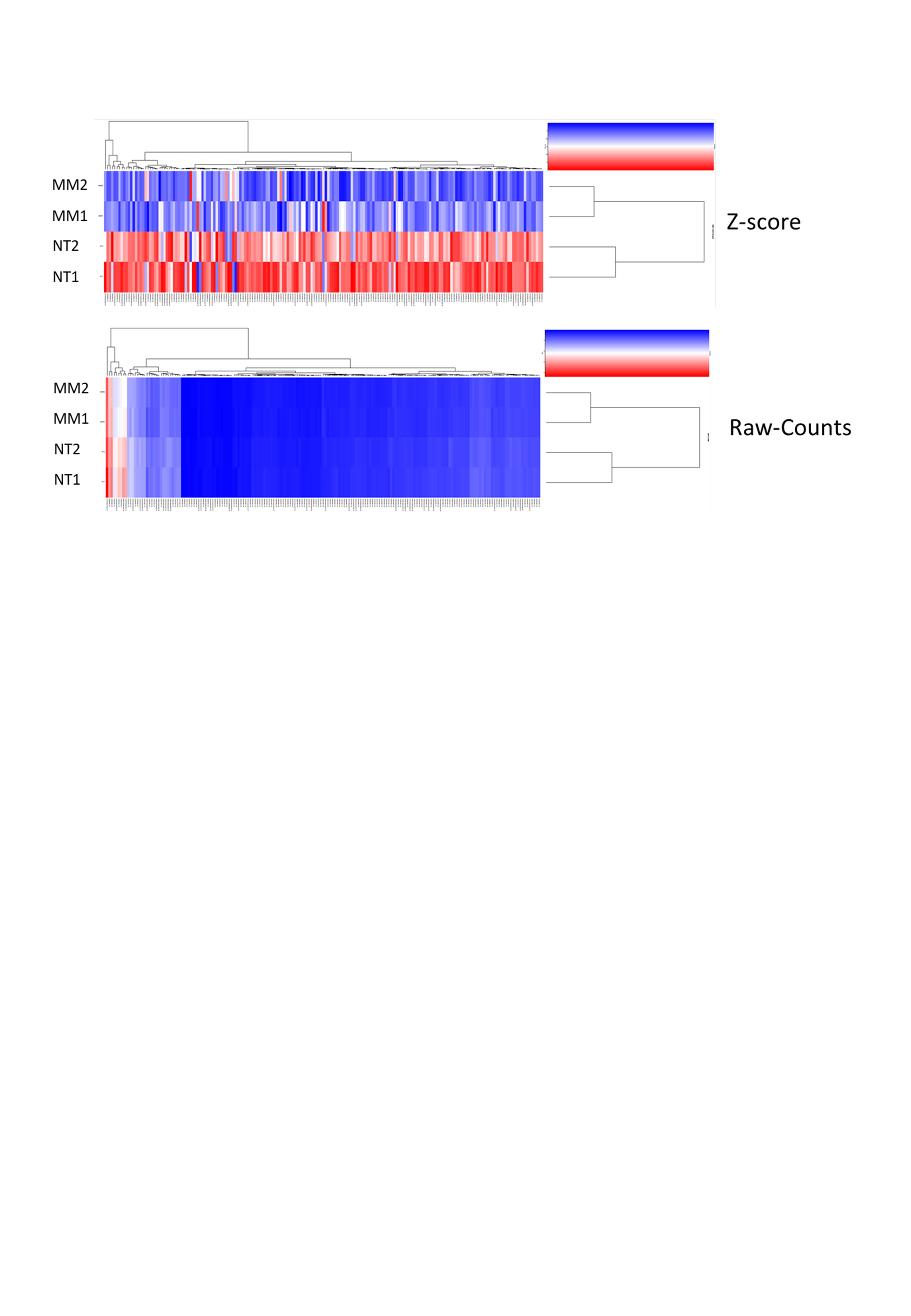

Supplement: Supplementary Figure 8 — Heatmap of gene-by-gene copy number variation in T. cruzi multigene families, in MMS treated MM (1-2) and untreated NT (1-2) TcTopo3α KO isolates. In this image, each line corresponds to an T. cruzi isolate, and each column corresponds to a gene. The copy number of each gene is represented in a scale from blue to red, denoting low and high counts, respectively. Columns (representing genes) and rows (representing samples) were clustered by UPGMA, based on the Manhattan distance of raw gene coverages. For each multigene family, two heatmaps were generated, one containing the read depth normalized by genome coverage (Raw-Counts), and one with the values normalized by Z-score by column. There was a significant reduction in counts on DGF-1 and RHS gene families, and a mild reduction in Trans-sialidases in MM1 and MM2, when compared with NT1 and NT2. [file Image_8.TIF]

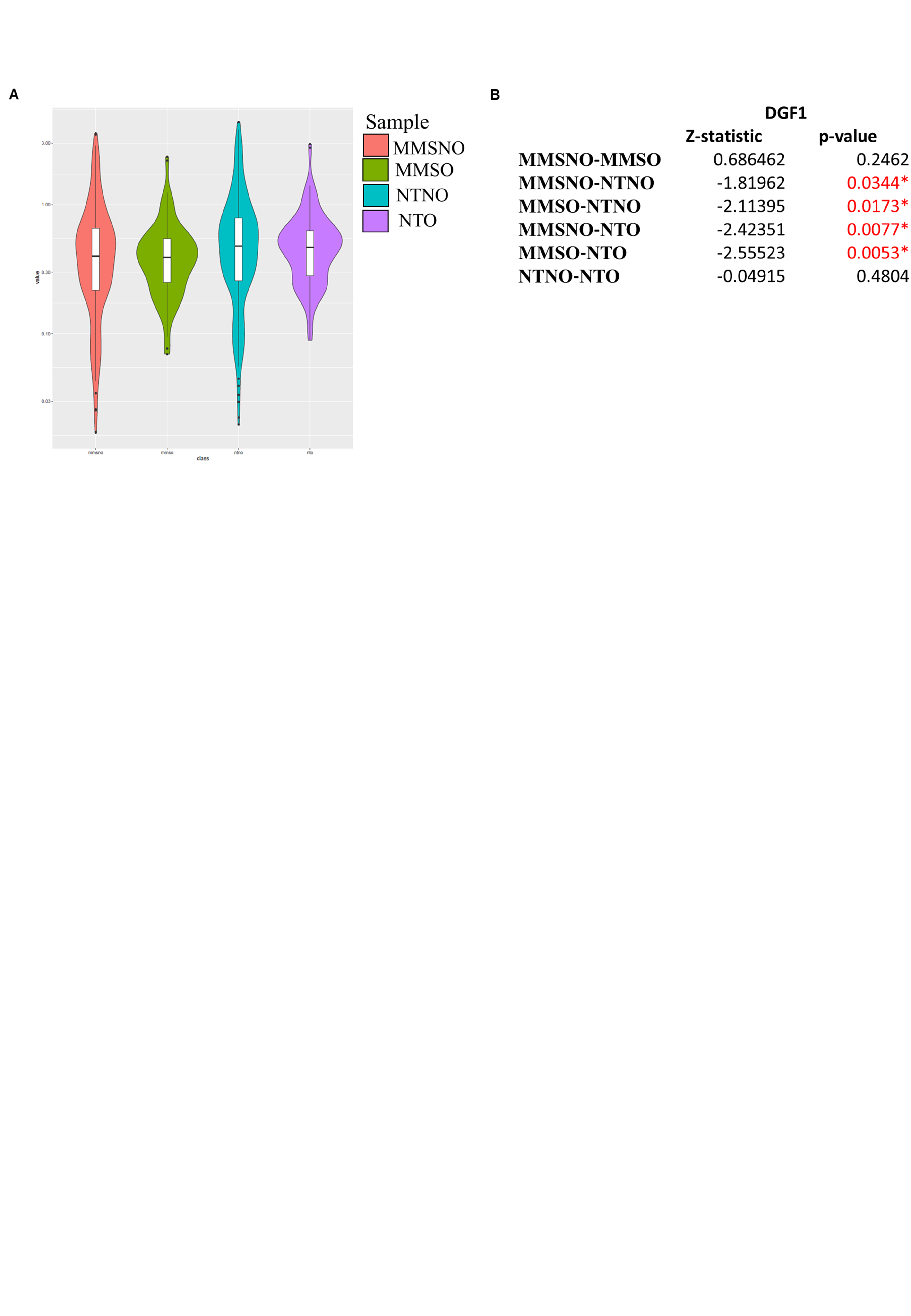

Supplement: Supplementary Figure 9 — Violin plot representing the gene copy number of DGF-1 genes near replication origins. (A) The Y axis represent the distribution of gene coverages, in log scale. MMS-treated DGF-1 non-origin (MMSNO – red), MMS-treated DGF-1 origin (MMSO – green), Non-Treated non-origin (NTNO – blue), and Non-Treated origin (NTO – purple). (B) Z-statistic and p-values of Dunn’s test. P-values < 0.05 are highlighted in red, and p-values ∼0.05 are highlighted in orange. There was no statistically supported difference in coverages of DGF-1 close or distant from replication origins (MMSNO-MMSO and NTNO-NTO). [file Image_9.TIF]

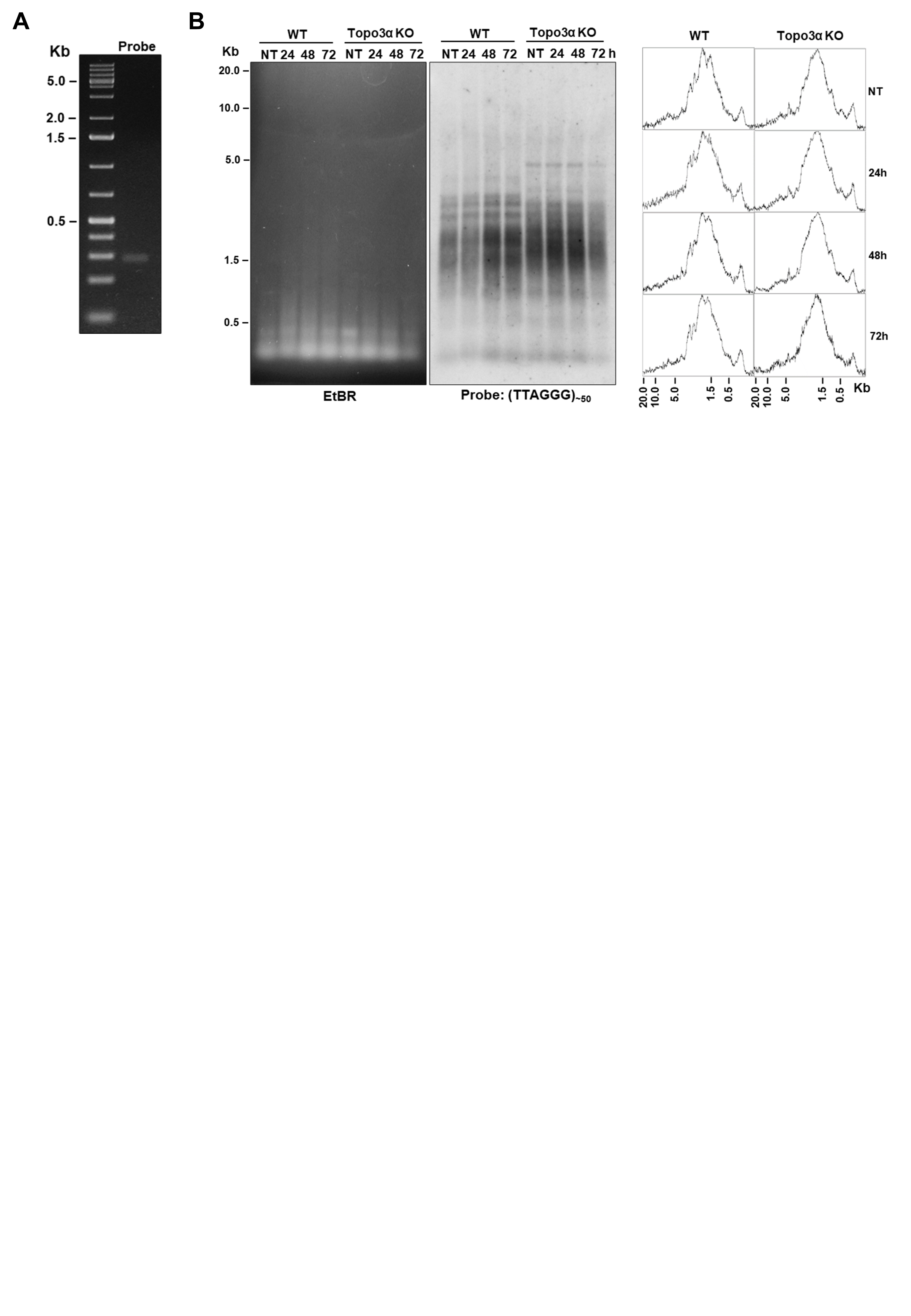

Supplement: Supplementary Figure 10 — Telomere length analysis by Southern blot. (A) Purified probe containing telomeric sequences repeat (TTAGGG)∼50 was generated by pTEL plasmid digestion with HindIII restriction enzyme. (B) Southern blot analysis of telomeric sequence-containing fragments generated by digestion of genomic DNA with restriction enzymes CviQI, HpaII, AluI, and HhaI; genomic DNA from WT and TcTopo3α KO cells was analyzed. Ethidium bromide-stained agarose gel showing the digestion products that were further analyzed by Southern blot. Right panel represents quantification of signal intensities for telomere hybridization using ImageJ software. Signals were plotted against DNA size (Kb). [file Image_10.TIF]

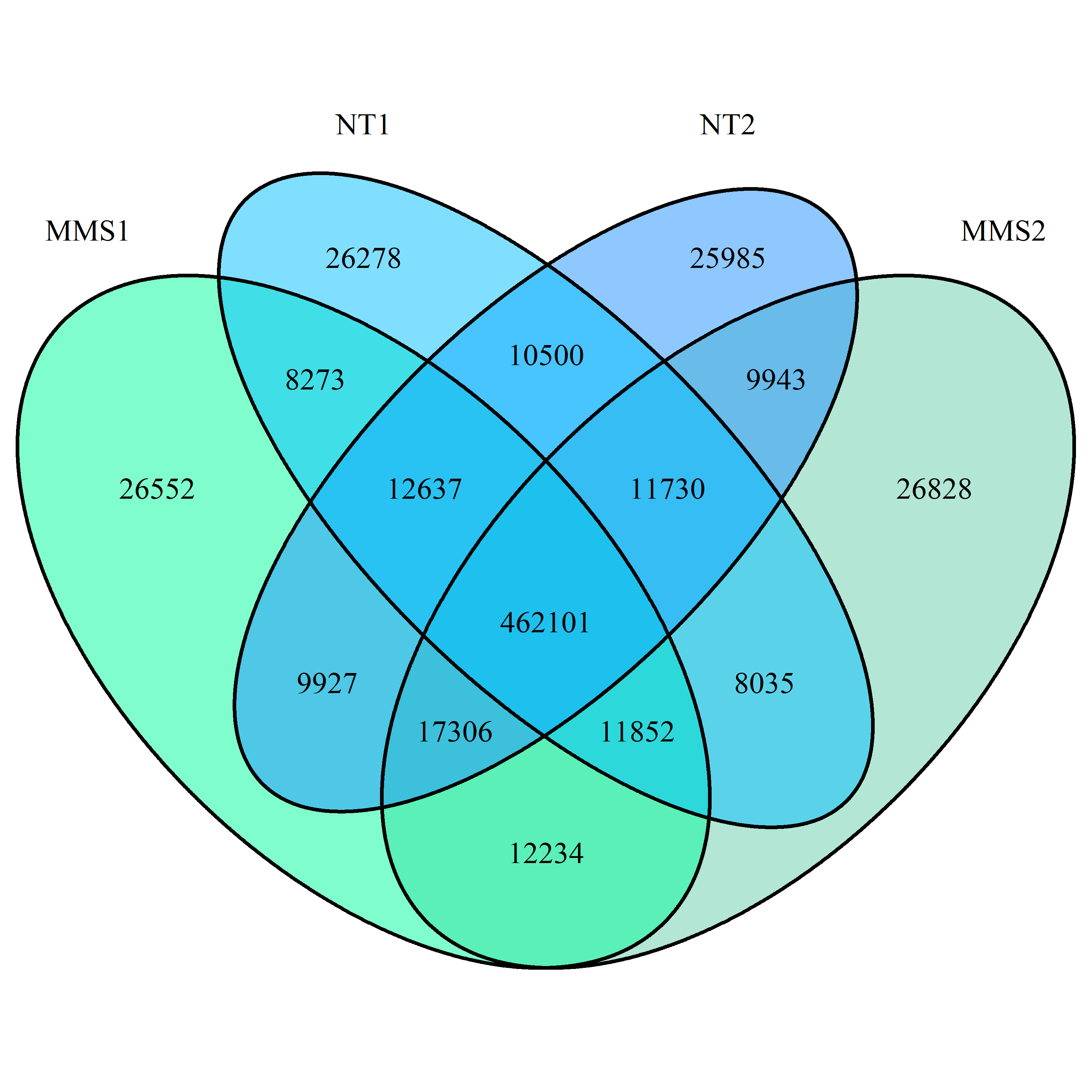

Supplement: Supplementary Figure 11 — Venn diagram of the SNPs in the MMS1, MMS2, NT1, and NT2 isolates. Venn diagram depicting the sharing of the SNP positions in the four T. cruzi evaluated isolates. [file Image_11.PNG]
